# Single-cell chromatin profiling of the primitive gut tube reveals regulatory dynamics underlying lineage fate decisions

**DOI:** 10.1038/s41467-022-30624-w

**Published:** 2022-05-26

**Authors:** Ryan J. Smith, Hongpan Zhang, Shengen Shawn Hu, Theodora Yung, Roshane Francis, Lilian Lee, Mark W. Onaitis, Peter B. Dirks, Chongzhi Zang, Tae-Hee Kim

**Affiliations:** 1 Program in Developmental & Stem Cell Biology, The Hospital for Sick Children, Toronto, Ontario M5G 0A4 Canada; 2 Department of Molecular Genetics, University of Toronto, Toronto, Ontario M5S 1A8 Canada; 3 Center for Public Health Genomics, University of Virginia School of Medicine, Charlottesville, VA USA; 4 Department of Biochemistry and Molecular Genetics, University of Virginia, Charlottesville, VA USA; 5 Division of Cardiovascular and Thoracic Surgery, University of California San Diego Medical Center, San Diego, CA USA; 6 Department of Public Health Sciences, University of Virginia, Charlottesville, VA USA

**Keywords:** Organogenesis, Endoderm, Epigenomics, Chromatin

## Abstract

Development of the gastrointestinal system occurs after gut tube closure, guided by spatial and temporal control of gene expression. However, it remains unclear what forces regulate these spatiotemporal gene expression patterns. Here we perform single-cell chromatin profiling of the primitive gut tube to reveal organ-specific chromatin patterns that reflect the anatomical patterns of distinct organs. We generate a comprehensive map of epigenomic changes throughout gut development, demonstrating that dynamic chromatin accessibility patterns associate with lineage-specific transcription factor binding events to regulate organ-specific gene expression. Additionally, we show that loss of Sox2 and Cdx2, foregut and hindgut lineage-specific transcription factors, respectively, leads to fate shifts in epigenomic patterns, linking transcription factor binding, chromatin accessibility, and lineage fate decisions in gut development. Notably, abnormal expression of Sox2 in the pancreas and intestine impairs lineage fate decisions in both development and adult homeostasis. Together, our findings define the chromatin and transcriptional mechanisms of organ identity and lineage plasticity in development and adult homeostasis.

## Abstract (web summary)

The primitive gut tube gives rise to all major internal organs, while underlying regulatory mechanisms are unclear. Here, the authors analyze its chromatin landscape at the single-cell level and define the epigenetic regulation of lineage fate decisions and plasticity in organ development and homeostasis.

## Introduction

In mammals, the primitive gut tube is the precursor to a number of critically important organs, which include the colon, intestine, stomach, pancreas, liver, lung, esophagus, pharynx, and thyroid^1^. The primitive gut tube forms at embryonic day (E) 8.5 as the left and right sides merge. This tube is mainly derived from two primary germ layers, with most of the cells being mesoderm-derived at this stage, which will give rise to the mesenchymal cells of the gut, such as fibroblasts, connective tissue, and blood vessels^2^. A single-cell thick layer of endodermal cells lines the inside of the primitive gut tube, which will go on to form the functional epithelial cells of the gut^2^. For example, these cells will form the absorptive enterocytes in the intestine, the acid-secreting parietal cells in the stomach, and the hormone-producing cells of the exocrine lineage in the pancreas^3–5^.

At E9.5, the first, subtle morphological changes take place. Regional domains for the intestine, stomach, liver, lung, and pancreas begin to bud off of the primitive gut tube in a process termed organogenesis; until E13.5, the morphological identities of these organs become increasingly defined. However, the endodermal layer remains uniform over this time, maintaining a single layer of pseudostratified epithelial cells. By E16.5, the organs of the gut have developed distinct boundaries between each other, and within themselves, establishing organ-specific functional roles for epithelial cells, a process known as regionalization^1,6^.

While the endodermal cells of the primitive gut tube (E8.5–E13.5) appear to be featureless, differences in gene expression are present along the anterior-posterior (AP) axis^7–9^. Recent evidence has elevated our understanding of spatial dynamics of gene expression patterns along the length of the primitive gut tube. Using single-cell (sc) RNA-seq, these studies created a comprehensive map of early transcriptional dynamics, demonstrating that organ fates are pre-programmed into cells at the earliest stage of gut development^10,11^. While these studies have provided unprecedented insight into the specificity of gene expression, it remains unclear what forces are governing this incredible spatial control over transcription.

Organogenesis and regionalization of the gastrointestinal tract have been the subject of intense investigation. Studies have revealed that several transcription factors (TFs) are expressed in specific organs and play a critical role in lineage fate decisions. For example, CDX2 is expressed specifically in the midgut and hindgut to control the intestinal fate^12–14^, while PDX1 regulates pancreatic identity and further contributes to the proximal intestine and the distal stomach fate^15–17^, and SOX2 demarcates the foregut, which will give rise to the stomach, esophagus, lung, pharynx, thymus, and thyroid^18,19^.

Recent studies examining chromatin accessibility and gene expression through next-generation sequencing in the developing stomach and intestine have begun to identify epigenetic changes occurring in a temporal- and region-specific manner^14,20,21^. While these studies revealed that a similar chromatin pattern exists between organs, they utilized bulk analyses as well as limited assessments to later stages of gut development, between E11.5 and E16.5. Importantly, organogenesis is well under way at the earliest stages that have been previously investigated. It is unclear whether organ-specific differences in chromatin accessibility precede organogenesis, or whether the organ-specific patterns of chromatin accessibility reflect transcriptional changes associated with developmental progression.

Here, we utilized recent advances in single-cell assay for transposase accessible chromatin with sequencing (scATAC-seq) to uncover patterns of chromatin accessibility in early gut development. Combining this single-cell analysis with bulk analysis from later developmental stages, we defined the spatial and temporal dynamics of chromatin accessibility across several organs. We further incorporated scRNA-seq and RNA-seq datasets to develop a comprehensive epigenetic landscape of gut organ development. Moreover, by performing mouse genetic studies in combination with epigenetic analysis, we demonstrated how lineage-defining TFs and chromatin accessibility regulate transcription and cooperatively define organ identity, as well as how their dysregulation contributes to altered organ functions and cell fates in development and adult homeostasis.

## Results

### Chromatin accessibility in the primitive gut tube predicts organ fate

**Fig. 1:**
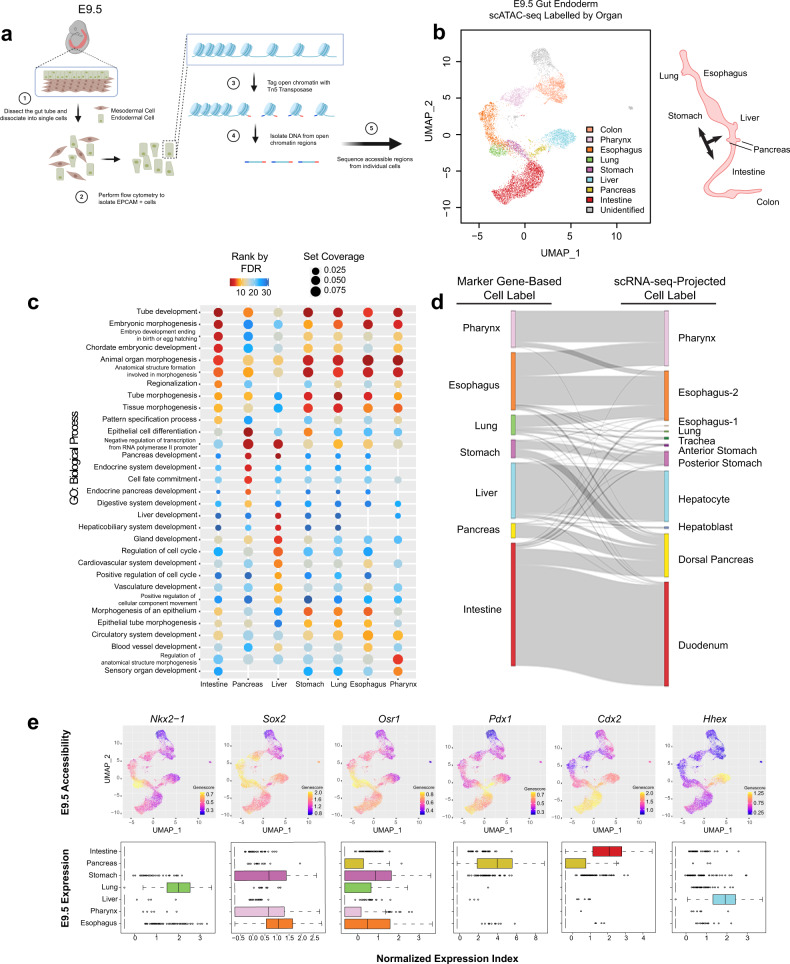
Chromatin accessibility predicts organ fate and functions in conjunction with transcription in the primitive gut tube. **a** Schematic diagram for the isolation of gut endodermal cells from E9.5 mice and scATAC-seq. **b** Single-cell scatter plot under UMAP dimensionality reduction demonstrating the clustering and organ assignment of cells. The organ assignment is conducted by comparing the gene score of known marker genes of organs. Each dot represents an individual cell and is colored by the assigned organ type. The spatial distribution pattern of organs on the UMAP resembles the actual anatomic positions of the organs on the right diagram. Black arrows label organ specification. **c** Organ-specific peaks for each organ cluster in (**b**) are assessed through a gene ontology of biological processes, with the top 10 processes of each organ displayed. GO terms are sorted in ascending order of their false discovery rate (FDR). Set coverage represents the fraction of all genes in the test set with the annotation. **d** Sankey diagram shows the relationship between marker gene-based organ labeling and scRNA-seq-projected organ labeling of cells from scATAC-seq data. **e** Known lineage-associated markers demonstrate organ-specific patterns of chromatin accessibility (top) and normalized expression (bottom). In UMAP scatter plots, cells are colored by gene score of the corresponding marker gene. In gene expression box plots (*N* = 9101, over 7 featured organs), the box centers represent median values, the lower and upper bounds of the boxes represent the first and third quantiles. The whiskers extend to the minimum and maximum values that do not exceed 1.5× IQR from the median values of the data (where IQR is the inter-quartile range).

To study the transcriptional regulatory patterns in the early gut development, we isolated Epithelial Cell Adhesion Molecule (EPCAM) expressing endodermal cells from the primitive gut tube through fluorescence-activated cell sorting at E9.5 for scATAC-seq (Fig. 1a, Figure S1a). Over two litters, we profiled the chromatin accessibility at the single-cell level and obtained 12,067 individual cells with high quality and reproducibility (Supplementary Dataset 1) (Figure S1b). We subsequently clustered cells based on their genome-wide chromatin accessibility patterns before visualizing the clusters through uniform manifold approximation and projection (UMAP) (Figure S1c, “Methods”). By summarizing chromatin accessibility signals located within 100 kb from each gene locus (“gene score”, “Methods”), we further grouped clusters together based on the “gene score” of known organ marker genes and generated a projection of organ fates (Fig. 1b). We noted a broad chromatin accessible pattern at *Sox2* across multiple clusters, consistent with its broad expression in foregut precursors^9^. To further resolve the foregut identities, we subclustered the *Sox2* accessible clusters by accessibility at *Pdx1* to identify the stomach and *Nkx2-1* to identify the lung (Figure S1d). Strikingly, we observed a near perfect match between the organ projection pattern on the scATAC-seq UMAP plot and the anatomical patterns of the organs that the primitive gut tube will develop (Fig. 1b, right).

We next asked whether our assigned cluster identities reflected the function of prospective organs. We identified chromatin accessibility peaks from each cell cluster with an assigned organ as a pseudo-bulk ATAC-seq sample and defined organ-specific peaks as those having significantly higher signals in that particular organ cluster compared with other cells. We performed a gene ontology (GO) analysis for biological processes to assess functions of each group of organ-specific peaks (“Methods”) (Fig. 1c). Notably, the accessibility patterns of specific peaks in each organ at E9.5 generally reflect the biological process that each organ will undergo later in development. For example, accessible regions in the prospective intestinal cells are associated with tube development, while pancreatic cells are associated with pancreatic development and endocrine system development, and liver cells are associated with both liver development and hepatobiliary development. These results strongly suggest that chromatin accessibility predicts lineage fate decisions in the primitive gut tube.

To understand how, mechanistically, chromatin accessibility patterns are capable of reflecting future organ-specific developmental processes, we further defined organ-specific accessibility peaks to explore the potential organ-specific binding events of TFs (Figure S2a). Recognizing that lineage fate decisions are driven by TF activity, we performed a DNA sequence motif analysis at differentially accessible chromatin to identify potential TFs that could explain the organ-specific enrichment of biological processes we have observed. Indeed, we were able to clearly identify organ-specific patterns of TF motifs and their family enrichment, such as CDX family in the intestine, SOX family in the stomach, and PDX family in the pancreas (Figure S2a, Supplementary Dataset 2). These data suggest that chromatin accessibility patterns associate with lineage-specific TF binding events, which program lineage fate decisions into individual cells of the early gut tube.

Ultimately, lineage fate decisions require transcriptional activation. By integrating our scATAC-seq data with scRNA-seq data from published E9.5 fore- and mid-gut endodermal cells^10^, we examined the relationship between patterns of chromatin accessibility and transcriptional output. Based on the existing organ assignments from the E9.5 scRNA-seq data (Figure S2b), we applied a “Reference Assembly Integration” method using Seurat^22^ and projected each cell from the scATAC-seq data to the scRNA-seq data and labeled the cell from scATAC-seq to its matched organ assigned to scRNA-seq (Figure S2c). We excluded the colon from this analysis, as the scRNA-seq data does not contain hindgut cells. Importantly, there was a strong overlap between the organs defined through scATAC-seq marker gene score and those through scRNA-seq projection (Fig. 1d); this integration analysis indicated a near perfect overlap in the intestine, pancreas, liver, and stomach, and a strong overlap in the esophagus, lung, and pharynx domains. Notably, key drivers of lineage fate, such as *Nkx2-1*^23^, *Sox2*^19,24^, *Osr1*^25^, *Pdx1*^16^*, Cdx2*^12–14^, and *Hhex*^26^, demonstrate both tissue-specific patterns of chromatin accessibility and transcription, which likely collaborate to drive lineage fate decisions (Fig. 1e, Figure S3). Together, we demonstrate that in the earliest stages of gut development, chromatin accessibility patterns with putative binding of lineage-specific TFs predict future lineage-fate decisions, revealing a relationship between chromatin accessibility, TF motifs, and transcriptional output which cooperate to lay the foundation of organ fates throughout the primitive gut tube.

### Chromatin accessibility reinforces lineage fate decisions over time

**Fig. 2:**
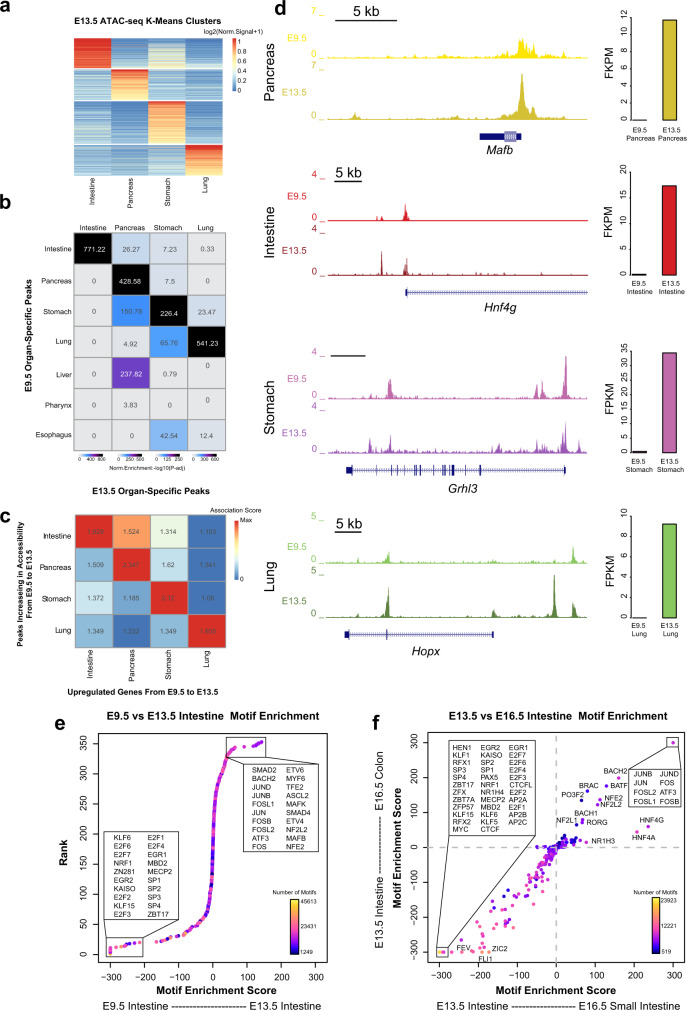
Temporal changes in transcription are reinforced by changes in chromatin accessibility at TF binding sites. **a** Heatmap of identified organ-specific peak clusters of bulk endodermal ATAC-seq samples from the E13.5 intestine, pancreas, lung, and stomach, colored by normalized ATAC-seq signal strengths. **b** Heatmap showing the significance of overlap between E13.5 organ-specific peaks and E9.5 organ-specific peaks. Values indicate the overlap significance quantified by −log10 (P-adj) using the one-sided hypergeometric test. **c** Heatmap of association scores (see “Methods” for details) between top 200 upregulated genes and top 2000 accessibility-increased peaks from E9.5 to E13.5 within and between each organ. **d** Example genes show the acquisition of lineage-specific patterns of chromatin accessibility (left) and transcriptional output (right) as development proceeds from E9.5 to E13.5. Y-axis on the genome-browser snapshots (left) represents normalized read count of pseudo-bulk (E9.5) or bulk (E13.5) ATAC-seq. Y-axis of the transcription barplots (right) represents normalized RNA-seq expression levels (FPKM) of E9.5 (scRNA-seq, *N* = 1153 cells) or E13.5 (bulk, *N* = 8 samples) organs. **e** DNA sequence motif enrichment in the open chromatin regions, ranked by relative motif enrichment scores (x-axis, see “Methods” for details) between E9.5 intestine and E13.5 intestine. 353 mouse TF motifs are included. Y-axis indicates the rank. The top 20 TFs on each side are labeled. Each data point is colored based on the total number of hits of TF’s motif in the open chromatin regions. **f** Scatter plot shows relative motif enrichment scores. The x-coordinate of each TF is its relative motif enrichment score calculated between the E13.5 intestine and E16.5 small intestine. The y-coordinate of each TF is its relative motif enrichment score calculated between E13.5 intestine and E16.5 colon. The top motifs on each end are labeled.

After gut tube closure, regional domains further develop into functionally distinct organs^1,6^. In vitro models of pancreatic differentiation using human pluripotent stem cells have shown that chromatin accessibility and TF bindings are dynamic over time, collaborating to establish and reinforce lineage fates decisions throughout development^27–29^. These findings raise the possibility that this phenomenon may hold true in other gut lineages, but this remains unknown. To address this question, we took an organ-by-organ approach, examining how chromatin accessibility and gene expression patterns change within organs over developmental time. In addition to utilizing our previously published bulk ATAC-seq data from epithelial cells of the intestine and stomach at E13.5^21^, we performed bulk ATAC-seq on epithelial cells of the E13.5 lung and pancreas, which allowed us to examine chromatin accessibility patterns in 4 major organs. We first determined organ-specific patterns of chromatin accessibility across the E13.5 tissues through K-means clustering (Fig. 2a). To understand how chromatin accessibility patterns change in each of these organs over time, we compared the organ-specific peaks among the 4 major organs in E13.5 to those defined in our E9.5 marker-based analysis. We observed a significant enrichment of organ-specific peaks between presumptive organ precursors at E9.5 and mid-development organs when we compared within organs over time, relative to comparisons between organs (Fig. 2b). These findings reinforce our idea that chromatin accessibility patterns in early gut tube formation reflect lineage fate decisions.

Within each organ, we next looked to determine whether changes in chromatin accessibility coincide with changes in transcription over time. To do so, we performed bulk RNA-seq on epithelial cells of the E13.5 stomach, intestine, lung, and pancreas, and identified tissue-specific transcripts through K-means clustering. This analysis produced an accurate list of organ-specific transcription patterns in the gut at E13.5 and excluded transcripts common to more than one organ (Figure S4). Within each organ, we then compared temporal (E9.5 to E13.5) changes between gene expression and chromatin accessibility (Supplementary Dataset 3). Specifically, for each of the 4 major organs, we selected the top 200 genes that increased expression between E9.5 and E13.5. Using these top gene lists, we examined the number of upregulated genes that also demonstrate an increase in chromatin accessibility between E9.5 and E13.5 (Supplementary Dataset 3). For the intestine, 31/200 (15.5%) of top upregulated genes also show an increase in accessibility, while 42/200 (21%) top upregulated genes in the pancreas also show this pattern. Likewise, 34/200 (17%) top upregulated genes in the stomach and 33/200 (16.5%) top upregulated genes in the lung also show increases in accessibility between E9.5 and E13.5. We further generated an “association score” to quantify the association compared to the genome-wide background (see “Methods”), and observed that the strongest associations between changes in transcription and accessibility over time occur within each given organ (Fig. 2c), as opposed to comparisons between organs over time. These data suggest that increases in chromatin accessibility highly correlate with transcriptional activation during development. Notable examples of corresponding transcriptional and accessibility changes include *MafB*^30^ in the pancreas, *Hnf4g* in the intestine^31^, *Grhl3* in the stomach^32^, and *Hopx* in the lung^33^ (Fig. 2d). While these examples are not necessarily tissue-specific, they represent the positive relationship between accessibility and transcription over developmental time (Figure S5).

Next, we identified peaks that are shared among foregut organs at E9.5 (stomach, lung, esophagus, thymus/thyroid). We further categorized these peaks based on their presence in the stomach and lung at E13.5 (Figure S6a). This allowed us to define patterns of lineage restriction at the level of chromatin accessibility. We then identified all genes commonly expressed in the foregut lineage at E9.5 and determined which of these become tissue specific by E13.5, allowing us to define lineage restriction of gene expression (Figure S6b, left). To understand the relationship between loss of chromatin accessibility and restriction of gene expression during early foregut development, we compared genes which become transcriptionally silent in one organ to those which experience a tissue-specific loss in chromatin accessibility in the same organ. Of the 511 genes which specifically became transcriptionally silent in the lung by E13.5, 51 (9.98%) of these genes experienced a tissue-specific loss in chromatin accessibility. In the developing stomach, 464 genes were specifically repressed by E13.5, yet 291 (62.72%) of these genes coincided with a loss of chromatin accessibility. This tissue-specific association between transcriptional silencing and loss of chromatin accessibility is significantly higher in the stomach relative to the lung (binomial test $p-{{{{{{\rm{value}}}}}}}=2.48\times {10}^{-137}$, Figure S6b right). This indicates that repression of gene expression is more strongly related to reduced chromatin accessibility in the stomach than the lung from E13.5 to E16.5. Examples of lineage restriction include *Nkx2-1* and *Rims4*, which, in both expression and chromatin accessibility, become restricted to the lung and stomach, respectively (Figure S6c, d).

In addition, we investigated how the association between transcriptional silencing and loss of chromatin accessibility of genes specific to the lung compares to genes common to both the stomach and lung. By E13.5, 47.27% of genes (286/605) common to the stomach and lung undergo both transcriptional silencing and lose chromatin accessibility, in contrast to 9.98% of lung-specific genes (binomial test $p-{{{{{{\rm{value}}}}}}}=4.03\times {10}^{-74}$, Figure S6b, right), which mirrors our comparison between lung-specific and stomach-specific genes. Collectively, our results describe the organ-specific association patterns between transcription and chromatin accessibility during the lineage restriction in foregut.

Although we have identified a strong relationship between chromatin accessibility and transcription as development progresses, it remains unclear how, mechanistically, these concurrent changes are orchestrated. We hypothesized that accessibility at binding sites of key TFs change throughout development, which may contribute to the maintenance or reinforcement of lineage fate decisions. Accordingly, we examined the genome-wide occurrence of TF binding motifs in organ-specific open chromatin regions and identified those TFs whose motifs occurred in the chromatin regions that exhibit differential accessibility between E9.5 and E13.5. In the intestine, we identified a number of TF families specifically accessible in E9.5 (E2F, KLF, ERG) and E13.5 (MAF, FOS, JUN) (Fig. 2e, x-axis). Among these, E2F^34,35^ and KLF^36^ family members have known roles in early embryonic intestinal development, while FOS and JUN members are known to be expressed in intestinal maturation^37^. To determine the relationship between chromatin accessibility at TF binding sites and the expression of the cognate TFs, we examined whether these TFs are transcriptionally active at E9.5 and E13.5. Interestingly, most of the TFs associated with differential accessibility had minor expression changes for their genes from E9.5 to E13.5 in any organ (Figure S7). However, there were notable examples of TFs with defined changes in both accessibility and expression over time. *Fos2l* showed E13.5-specific binding site accessibility and expression in the intestine, while *Gata3* was specifically expressed and accessible at binding sites in the lung at E9.5. In addition, the stomach showed E9.5-specific patterns of expression and binding site accessibility for *Gata2*, *Gata3*, and *Gata6*, as well as E13.5-specific expression and binding site accessibility of *Fos2l*, *Fosb*, and *Fos* (Figure S7).

We then questioned whether chromatin accessibility patterns continue to change into late organ development. To address this question, we made use of the publicly available bulk ATAC-seq data from the epithelial cells of the forestomach, hindstomach, small intestine, and colon at E16.5^20,21^, and performed a similar motif enrichment analysis (Fig. 2f, Figure S8). Through this analysis, we were able to characterize the binding sites specifically associated with mid (E13.5) and late (E16.5) stage organ development. In all tissues we observed a strong enrichment of FOS and JUN family members specifically at E16.5, suggesting that this pathway may play an integral role in tissue maturation and growth^37,38^. Conversely, we found enrichment of SP, KLF, and AP2 family members at E13.5, suggesting that these factors may play a more important role earlier in development (Fig. 2f, Figure S8). In the intestinal samples, we also found enrichment of HNF4α and HNF4γ in the late-stage colon and small intestine, with a stronger trend toward small intestinal identity compared to the colon. This outcome was consistent with the known function of HNF4α as a key regulator intestinal epithelial differentiation and absorption^31^. Similar to our results from E9.5 to E13.5, changes in accessibility at TF binding sites showed little relationship to the expression of these TFs (Figure S8), suggesting that developmental trajectories in the gut are linked to chromatin accessibility at TF binding sites. By combining scATAC-seq, bulk ATAC-seq, and bulk RNA-seq, we demonstrate that each organ in the developing gut uses its own suite of regulatory machinery to push forward its developmental trajectory.

### Transcription factors cooperate with chromatin accessibility to regulate cell fate decisions

Our data indicate a strong cooperative relationship between chromatin accessibility, TF binding, and transcription in the regulation of lineage fate decisions in the developing gut, which begins at the earliest stage of gut development and persists over time. A number of organ-specific TFs are known to be critical for maturation and differentiation of epithelial cells in the developing gut. For example, SOX2 and CDX2 represent mutually exclusive and adjacent domains, regulating development of the foregut (stomach, lung, esophagus, pharynx) and hindgut (small intestine, colon), respectively^9^. While we have provided evidence that dynamic chromatin accessibility may be associated with binding of such organ-specific TFs, we sought to understand how these TFs interact with chromatin to regulate organ fate in the developing gut.

**Fig. 3:**
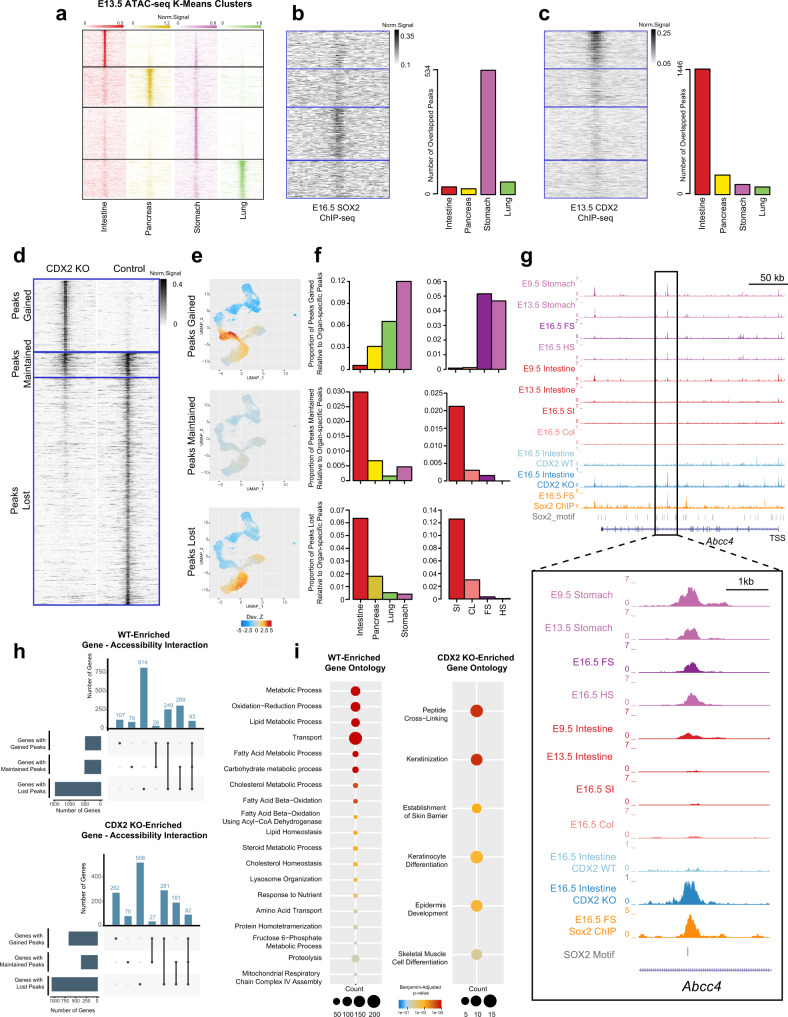
SOX2 and CDX2 binding sites are associated with lineage specification patterns of chromatin accessibility. **a** ATAC-seq signal pattern on E13.5 organ-specific peaks in the intestine, pancreas, stomach, and lung. Peaks are ordered by K-means clustering of ATAC-seq signal in the 4 organs. Color scale represents normalized ATAC-seq signals. **b**, **c** SOX2 ChIP-seq (*n* = 2) signal pattern in E16.5 stomach (**b**) and CDX2 ChIP-seq (*n* = 2) signal pattern in E13.5 intestine (**c**), aligned to the same region as (**a**) (left). Gray scale represents normalized ChIP-seq signals. Barplots showing numbers of organ-specific ATAC-seq (*N* = 7 ) peaks overlapped with SOX2 (**b**) or CDX2 (**c**) binding sites (right). **d** ATAC-seq signal pattern around genome-wide chromatin accessibility peaks in the E16.5 intestine from *Cdx2* KO (left) and control (right) samples. Peaks are grouped into three categories based on how chromatin accessibility changes upon *Cdx2* KO: Gained (top), Maintained (middle), and Lost (bottom). Gray scale represents normalized ATAC-seq signals. **e** Single-cell scatter plots under the same UMAP representation of E9.5 scATAC-seq as Fig. 1b, with each cell colored by its ChromVAR deviation Z-score for *Cdx2* KO Peaks Gained, Maintained, or Lost. **f** Proportion of chromatin accessibility peaks Gained, Maintained, or Lost upon *Cdx2* KO that are overlapped with E13.5 organ-specific chromatin accessibility peaks in the intestine, pancreas, lung, and stomach (*N* = 7, left) and with E16.5 organ-specific chromatin accessibility peaks in the small intestine (SI), colon (CL), forestomach (FS), and hindstomach (HS) (*N* = 8, right). **g** Genome browser snapshot at the *Abcc4* locus depicting chromatin accessibility at a SOX2 binding site in the E9.5 and 13.5 stomach and intestine, the E16.5 SI, CL, FS, and HS, and the E16.5 control and *Cdx2* KO intestine. **h** UpSet plot showing the intersections of WT (*n* = 3, top) and *Cdx2*-KO (*n* = 3, bottom) differentially expressed genes associated with different peak categories. **i** Enriched biological process GO terms of WT-specific genes with lost peaks (left) and *Cdx2*-KO-specific genes with gained peaks (right). Bubble size represents number of genes; Color represents significance based on Benjamini–adjusted *P*-value of one-sided fisher exact test from DAVID GO analysis.

Utilizing existing SOX2 and CDX2 ChIP-seq data generated in the E16.5 stomach and E13.5 intestine, respectively^14,21^, we examined how the binding of these key lineage-specific TFs corresponds to organ-specific patterns of chromatin accessibility. We first compared the organ-specific accessibility peaks that we previously identified from each of the four organs at E13.5 (Fig. 3a) to SOX2 and CDX2 ChIP-seq signals and counted the number of overlapping ChIP-seq and accessibility peaks per organ. It was clear that SOX2 binding in the stomach specifically overlaps with accessible regions specific to the stomach (Fig. 3b), while CDX2 binds to accessible regions specific to the intestine (Fig. 3c), suggesting that chromatin accessibility patterns may indicate simultaneous or future binding patterns of organ-specific TFs that regulate lineage fate decisions.

Knowing that organ-specific patterns of chromatin accessibility reflect SOX2 and CDX2 binding, we wondered whether the binding of these factors would be essential to establish chromatin accessibility and define organ identity. To address this question, we examined existing bulk ATAC-seq data from E16.5 *Sox2* knockout (KO) stomach^21^ and *Cdx2* KO intestine samples^14^ and observed drastic changes in chromatin accessibility patterns associated with loss of either TF (Fig. 3d, Figure S9a). We grouped accessibility peaks into three distinct categories based on the ATAC-seq signal changes between KO and Control samples: peaks gained upon loss of the TF (top), peaks that remain unchanged (middle), and peaks that are lost (bottom). We mapped the peaks from each category onto the scATAC-seq data from E9.5, quantified the relative enrichment of chromatin accessibility levels in the peak regions in each cell compared to all cells using chromVAR^39^ (see “Methods”), and marked the cell-level enrichment on the scATAC-seq UMAP (Fig. 3e, Figure S9b).

In the *Sox2* KO stomach, we observed that gained peaks are strongly associated with the chromatin accessibility pattern of the cell clusters projected as stomach and lung, and that maintained peaks are associated with the projected lung, stomach, pancreas, and small intestine clusters, whereas lost peaks are not strongly associated with any cluster (Figure S9b). This finding suggests that loss of *Sox2* leads to an acquisition of early foregut identity, particularly the lung. To assess whether the changed accessibility peaks may associate with altered organ identity during development, we compared groups of peaks gained, maintained, and lost to our E13.5 and E16.5 bulk ATAC-seq datasets, examining the proportion of overlapping peaks between peaks gained, maintained, and lost relative to the organ-specific peaks from each organ (Figure S9c). Interestingly, gained peaks were strongly associated with lung identity at E13.5, whereas lost peaks were associated with stomach identity, suggesting a fate shift from stomach to lung. At E16.5, we continued to observe a loss of stomach identity, specifically hindstomach fate, and a concurrent acquisition of intestinal identity (both colon and small intestine), as well as a forestomach fate (Figure S9c). Collectively, analysis of accessibility peaks in *Sox2* knockout stomachs reveal a consistent loss of mature gastric fate, shifting chromatin accessibility patterns to be more similar to neighboring organs such as the lung and, to a smaller extent, the intestine.

We next performed a similar series of analyses for *Cdx2* KO intestinal epithelial cells and examined how the changed accessibility patterns upon loss of this TF associate with organ fate decisions throughout gut development. At E9.5, we found a striking shift in chromatin accessibility patterns with gained peaks associated with foregut clusters and lost peaks associated exclusively with intestinal cells (Fig. 3e). This dramatic shift in chromatin accessibility away from intestinal fate towards foregut fates was maintained at both E13.5 and E16.5 (Fig. 3f), confirming that CDX2 maintains chromatin accessibility patterns associated with intestinal identity^14,20^. Accordingly, *Cdx2* deletion shifts chromatin accessibility patterns away from the intestine to strongly resemble foregut accessibility patterns. For example, *Abcc4* is a gastric-specific gene, which is consistently accessible at a SOX2 binding site throughout development. *Cdx2* deletion induced a specific increase of accessibility at this exact SOX2 binding site, concurrent with an acquisition of gastric identity (Fig. 3g).

Finally, we examined whether the shifts in lineage fates we observe at the chromatin level reflect changes in lineage fates at the gene expression level. To address this question, we made use of publicly available *Cdx2* KO and control intestinal microarray data^12^ (Supplementary Dataset 4) and compared the *Cdx2* KO-specific genes to the *Cdx2* KO chromatin accessibility peak lists (peaks gained, maintained, and lost), while performing the same analysis with control samples (Fig. 3h). Of the 2173 *Cdx2* KO-specific genes, 262 (12.1%) are associated with accessibility peaks gained upon *Cdx2* loss uniquely, while 508 (23.4%) are associated with peaks lost uniquely. In contrast, of the 2222 WT-specific genes, only 107 (4.8%) are associated with gained peaks uniquely, while 814 (36.6%) are associated with lost peaks uniquely (Fig. 3h). These data demonstrate that genes which become active in the *Cdx2* KO intestine are more likely (binomial test $p-{{{{{{\rm{value}}}}}}}=5.02\times {10}^{-21}$) to be associated with gained accessibility peaks resulting from *Cdx2* loss when compared to genes specifically active in the WT intestine. Instead, genes specific to the WT intestine are more strongly associated with peaks lost in the *Cdx2* KO. Together, these results indicate that loss of *Cdx2* drives a shift in chromatin accessibility away from intestinal fate, which coincides with alterations in gene expression.

To understand the functional consequence of this shift, we took all *Cdx2*-specifc genes (total 652 genes) that have an increase in chromatin accessibility, regardless of their association with maintained or lost peaks, and performed a GO analysis (Supplementary Dataset 5). Strikingly, we observed a strong enrichment of genes involved in keratinocyte differentiation (*P* = 9.3 × 10^−6^), indicating that loss of *Cdx2* leads to acquisition of a chromatin accessibility and transcriptional landscape reminiscent of the keratinized foregut. In contrast, performing the same GO analysis with WT-specific genes shows an enrichment of many metabolic and transportation processes, which are critical to normal intestinal function (Fig. 3i). Importantly, these reflect previous work, which has shown that loss of *Cdx2* induces a fate change in the intestine, leading to the acquisition of squamous, keratinized epithelium and loss of intestinal fate^12–14,20,21^.

### Misexpression of Sox2 in gut precursors alters organ identity

Previously, we and others showed that loss of key lineage-specific transcription factors in the stomach and intestine alters patterns of chromatin accessibility and gene expression, ultimately leading to changes in cell identity^14,20,21,31,40,41^. Specifically, *Cdx2* deletion induced SOX2 in the intestinal epithelium and activated stomach-like patterns of chromatin accessibility, concurrent with an acquisition of squamous keratinized epithelial cells typically found in the murine forestomach and esophagus^12,21^. However, *Sox2* overexpression after organ formation still maintained intestinal identity and morphology^42^. Interestingly, various phenotypes were observed upon *Cdx2* deletion at different embryonic stages, suggesting developmental plasticity^14^. Therefore, it remains unclear if *Sox2* expression alone at earlier developmental stages could sufficiently alter organ identity.

**Fig. 4:**
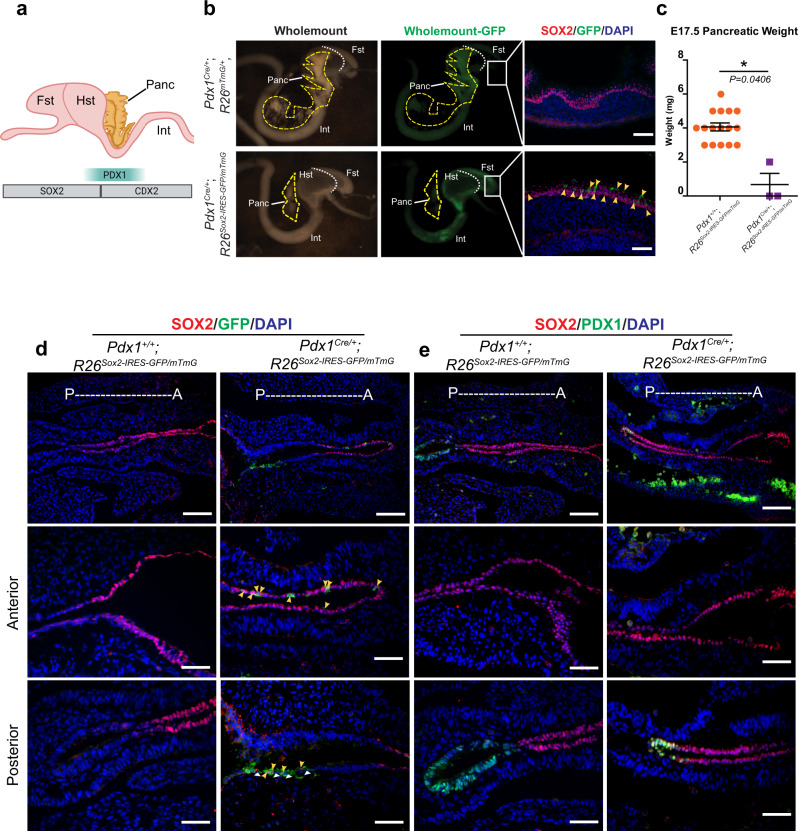
Embryonic overexpression of *Sox2* alters lineage fate decisions in the pancreas. **a** Schematic overview of SOX2, PDX1, and CDX2 expression in the developing forestomach (Fst), hindstomach (Hst), intestine (int), and pancreas (Panc). **b** Whole-mount light (left) and GFP (middle) images of E17.5 *Pdx1*^*Cre/+*^;*R26*^*mTmG/+*^ (*n* = 3, top) and *Pdx1*^*Cre/+*^;*R26*^*Sox2-IRES-GFP/mTmG*^ mice (*n* = 3, bottom) with GFP (green) and SOX2 (red) immunofluorescence images of Fst tissue (right). White dotted line denotes the boundary between Fst and Hst. Yellow dashed lines outline the pancreas. Yellow arrowheads indicate GFP-expressing cells in the Fst. Scale bar = 50 µm. **c** Mean (±SEM) pancreas weight (mg) from E17.5 *Pdx1*^*+/+*^;*R26*^*Sox2-IRES-GFP/mTmG*^ (*n* = 16) and *Pdx1*^*Cre/+*^;*R26*^*Sox2-IRES-GFP/mTmG*^ mice (*n* = 3) (*P *=* 0.0406 using a two-sided, unpaired t-test with Welch’s Correction). Source data are provided as a source data file. **d** Immunofluorescence images of SOX2 (red) and GFP (green) in E9.5 *Pdx1*^*+/+*^;*R26*^*Sox2-IRES-GFP/mTmG*^ (*n *= 3, left) and *Pdx1*^*Cre/+*^;*R26*^*Sox2-IRES-GFP/mTmG*^ (*n* = 3, right) mice. The top row shows both anterior and posterior domains (scale bar = 100 µm), while the middle and bottom rows show the anterior and posterior domains, respectively (scale bar = 50 µm). Yellow arrowheads indicate SOX2 and GFP double-positive cells. White arrowheads indicate GFP positive, SOX2 negative cells. **e** Immunofluorescence images of SOX2 (red) and PDX1 (green) in E9.5 *Pdx1*^*+/+*^;*R26*^*Sox2-IRES-GFP/mTmG*^ (*n* = 3, left) and *Pdx1*^*Cre/+*^;*R26*^*Sox2-IRES-GFP/mTmG*^ (*n* = 3, right) mice. The top row shows both anterior and posterior domains (scale bar = 100 µm), while the middle and bottom rows show the anterior and posterior domains, respectively (scale bar = 50 µm).

Our chromatin and gene expression analysis of the gut endoderm (E9.5) identified *Pdx1* as a key branching point TF, which labels the entire pancreas, the distal stomach, and the most proximal intestine, but its expression was largely excluded from the remaining organs (Fig. 4a)^15–17^. Importantly, *Sox2* expression was restricted to the foregut; neither the pancreas nor intestine expressed *Sox2*. Therefore, we generated a mouse model (*Pdx1*^*Cre/+*^;*R26*^*Sox2-IRES-GFP*^), targeting *Sox2* expression to the hindstomach (where it is excluded in late gut development), the intestine (to reflect observed SOX2 expression in *Cdx2* knockout mice), and the pancreas.

Surprisingly, *Sox2* overexpression led to a dramatic loss of pancreatic mass or even the complete absence of the pancreas (Fig. 4b, c). We next confirmed that *Sox2* was indeed overexpressed in these mice, noting that SOX2 protein is present in the distal stomach and intestine (Figure S10). Interestingly, we found a conspicuous absence of SOX2 expression in the remaining pancreas, implying that SOX2-expressing pancreatic precursors might have obtained a different organ fate (Figure S10a). To determine whether *Sox2* overexpression may influence lineage-fate decisions, we performed lineage-tracing on PDX1-derived cells (*Pdx1*^*Cre/+*^;*R26*^*Sox2-IRES-GFP/mTmG*^). Given that PDX1 expression is restricted to the proximal intestine, pancreas, and distal hindstomach, we would expect the membrane GFP (*mTmG*) expression to be restricted to these domains, as it is in control mice. However, we found ectopic GFP expression in the forestomach, suggesting that pancreatic progenitors, originating from the PDX1 domain, indeed have been transformed into forestomach cells (Fig. 4b).

Closer examination of the remaining pancreatic tissue revealed no changes in fate or identity. Since *Sox2* was not properly expressed (Figure S10a), we hypothesized that overexpression of *Sox2* may reduce the number of pancreatic precursors and only those that escape CRE-mediated *Sox2* expression produce differentiated pancreatic cells. To address this question, we analyzed mutant mice overexpressing *Sox2* at E9.5. Notably, *Sox2* overexpression in *Pdx1* lineage-derived cells induced a dramatic fate shift of pancreatic precursors. We observed that GFP-expressing cells are clearly expressed in the foregut domain (anterior), completely separated from the pancreatic domain (posterior) (Fig. 4d). While these cells expressed SOX2, they no longer expressed PDX1, further supporting pancreatic transformation into foregut fates (Fig. 4e).

In the small intestine, SOX2 expression was limited to small pockets of cells in the duodenum. Structurally, these groups of cells were disorganized, losing the characteristic “finger-like” projections into the lumen, and possessing instead cyst-like structures (Figure S10b). This structure coincided with a loss of intestinal identity, specifically loss of TFF3 (marking the mucous-secreting goblet cells) and CDX2 (marking all intestinal epithelial cells). These data show that intestinal overexpression of *Sox2* at E9.5 results in a loss of intestinal fate (Figure S10b). Together, our data demonstrate that *Sox2* overexpression in the developing gut endoderm is sufficient to impair intestinal lineage fate decisions, and to completely transform a pancreatic cell fate into foregut cell fates.

### Abnormal SOX2 expression in adult intestinal and pancreatic epithelial cells impairs their lineage fate decisions

**Fig. 5:**
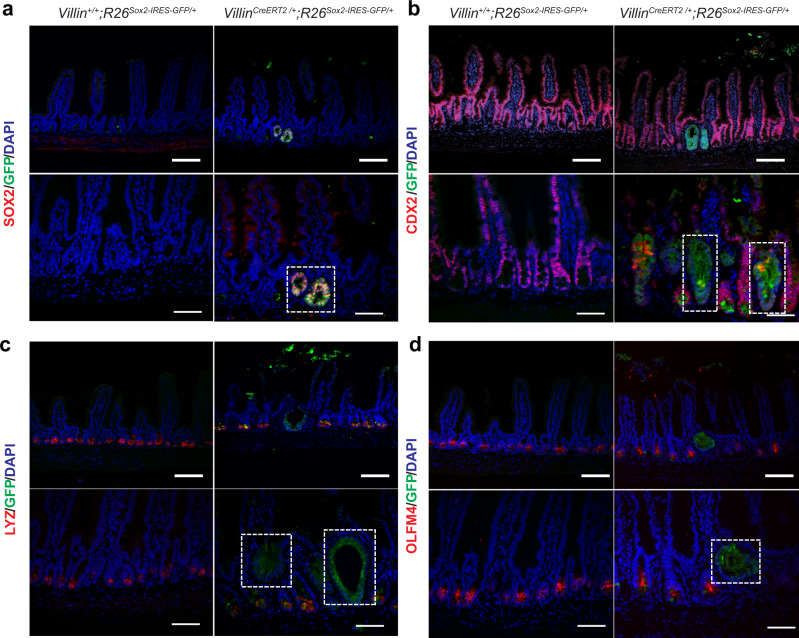
*Sox2* overexpression impairs intestinal identity in adult homeostasis. **a**–**d** Immunofluorescence images of *Villin*^*+/+*^;*R26*^*Sox2-IRES-GFP +*^ (*n* = 3, left) and *Villin*^*CreERT2/+*^;*R26*^*Sox2-IRES-GFP*^ (*n* = 4, right) for **a** SOX2 (red) and GFP (green), **b** CDX2 (red) and GFP (green), **c** Lysozyme C (LYZ, red) and GFP (green), and **d** OLFM4 (red) and GFP (green). Scale bars of **a**–**d** are 100 µm for top panels and 50 µm for bottom panels. White dotted boxes indicate areas of GFP expression.

Notably, a foregut lineage-specific TF, *SOX2*, which is not expressed in the intestine and pancreas, has been shown to be abnormally activated in colon and pancreatic cancers^43,44^. To understand a relationship between changes in organ identity and chromatin-mediated TF activities in adult homeostasis, we developed a mouse model to conditionally target *Sox2* expression to all intestinal epithelial cells (*Villin*^*CreERT2/+*^;*R26*^*Sox2-IRES-GFP*^) (Figure S11a). Eight weeks after tamoxifen treatment, we observed no obvious morphological phenotypes in the intestinal villus architecture (Figure S11b). However, we found small areas of GFP and SOX2 double-positive cells residing at the crypt base (Fig. 5a). Interestingly, these cells no longer expressed markers of intestinal fate, which include CDX2 (all intestinal epithelial cells) (Fig. 5b), Lysozyme C (Paneth cells) (Fig. 5c), OLFM4 (intestinal stem cells) (Fig. 5d), or TFF3 (goblet cells) (Figure S11c). These data demonstrate that prolonged *Sox2* expression is sufficient to impede intestinal epithelial differentiation in adult homeostasis.

Interestingly, cell fate changes occur as an early step in pancreatic cancer development; hormone-producing acinar cells will undergo a fate shift towards ductal cells in a process known as acinar-ductal metaplasia (ADM)^45^. Given time, this process leads to the development of a precancerous lesion known as a pancreatic intraepithelial neoplasia (PanIN)^46^. Therefore, we hypothesized that chronic activation of *Sox2* in adult pancreatic epithelial cells would be sufficient to drive fate changes and tumor-related phenotypes. To that end, we developed a mouse model that harbors a *Mist1*^*CreERT2/+*^ allele^47^ to target *Sox2* expression to epithelial cells of the pancreas (*Mist1*^*CreERT2/+*^;*R26*^*Sox2-IRES-GFP+*^). By performing a lineage tracing analysis (*Mist1*^*CreERT2/+*^;*R26*^*mTmG/+*^), we first confirmed that *Mist1*^*CreERT2/+*^ mice target acinar cells of the pancreas (Figure S12a, b). *Sox2* activation in acinar cells led to the development of small, misshapen duct-like structures visible in histological analyses (Figure S12c). Interestingly, these rare structures seemed to produce no amylase, despite being derived from acinar cells (Figure S12d). While we observed clear activation of SOX2 throughout the pancreas, there was no alteration in proliferation (Figure S12e, f).

**Fig. 6:**
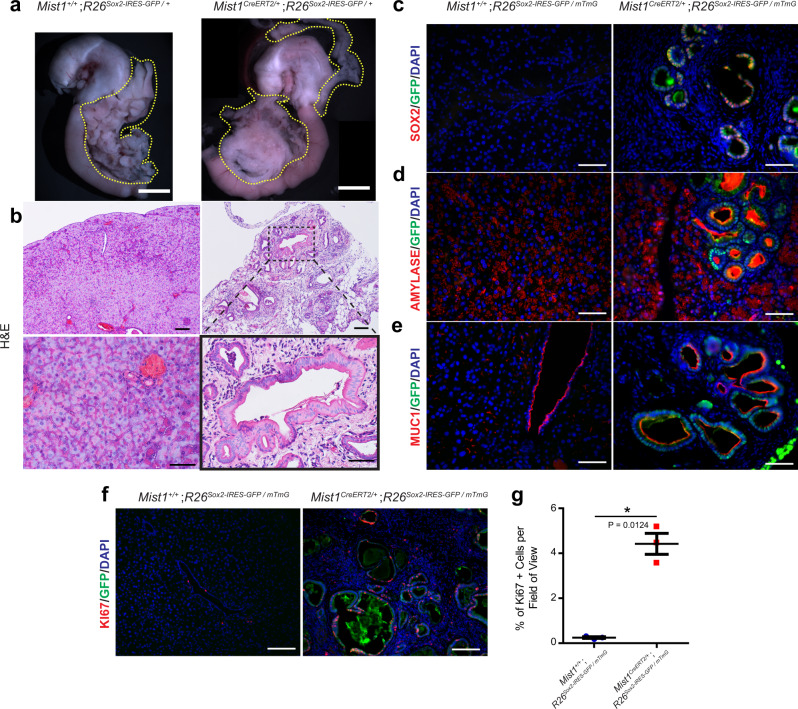
*Sox2* overexpression in adult pancreatic epithelial cells drives acinar-ductal metaplasia. **a** Whole-mount images of *Mist1*^*+/+*^;*R26*^*Sox2-IRES-GFP/+*^ (*n* = 3, left) and *Mist1*^*CreERT2/+*^;*R26*^*Sox2-IRES-GFP/+*^ (*n* = 3, right). Scale bar = 5 mm, yellow dotted lines outline the pancreas. **b** H&E staining of *Mist1*^*+/+*^;*R26*^*Sox2-IRES-GFP/+*^ (left) and *Mist1*^*CreERT2/+*^;*R26*^*Sox2-IRES-GFP/+*^ (right) pancreas tissue. Scale bars are 100 µm for top panels and 50 µm for bottom panels. Black dashed box represents the magnified area. Immunofluorescence staining of *Mist1*^*+/+*^;*R26*^*Sox2-IRES-GFP/+*^ (left) and *Mist1*^*CreERT2/+*^;*R26*^*Sox2-IRES-GFP/+*^ (right) pancreas for **c** SOX2 (red) and GFP (green), **d** Amylase (red) and GFP (green), **e** MUC1 (red) and GFP (green), and **f** Ki67 (red) and GFP (green). Scale bars for (**a**–**f**) are 50 µm. **g** Mean (±SEM) percent of Ki67 positive cells in *Mist1*^*+/+*^;*R26*^*Sox2-IRES-GFP/+*^ (left, *n* = 3) and *Mist1*^*CreERT2/+*^;*R26*^*Sox2-IRES-GFP/+*^ (right, *n* = 3) pancreas tissue (*P = 0.0124, by two-sided, unpaired *t*-test with Welch’s correction). Source data are provided as a source data file.

To next examine the effect of prolonged *Sox2* activation in pancreatic tissue, we induced *Sox2* expression and analyzed mice 8 weeks after induction (Fig. 6a, Figure S12a). Upon dissection, we found a clear alteration in pancreatic morphology. Both dorsal and ventral pancreatic domains appeared to have lost its characteristic branching structure, presenting instead as a disordered mass (Fig. 6a). Histological examination of these mice revealed substantial changes to morphology throughout the pancreas: a dramatic loss (but not elimination) of acinar cells, large amounts of immune cell infiltration, and a large number of cyst-like structures scattered throughout the tissue (Fig. 6b), reminiscent of the structures we noticed in the short-term experiment (Figure S12c–e).

We reasoned that *Sox2* expression might cause our observed phenotypes, so we first assessed SOX2 and GFP expression. Strikingly, we found that SOX2 and GFP are specifically expressed in these cyst-like structures, despite being first activated in acinar cells (Fig. 6c). We then examined whether SOX2 expression leads to a change in cell fate. We found that the SOX2-expressing cyst-like cells were unable to produce amylase in a manner similar to acinar cells, but large amounts of amylase were concentrated in the center of these structures (Fig. 6d). Since ductal cells are responsible for shuttling amylase out of the pancreas^48^, we wondered if these SOX2+ cells had shifted from acinar cells to ductal cells. Examination of MUC1^49^ revealed that SOX2+ cells are indeed ductal cells (Fig. 6e), clearly demonstrating that prolonged SOX2 exposure is sufficient to drive ADM in differentiated adult pancreatic cells. In addition, these pancreatic tissues appeared to be hyperproliferative, as marked by Ki67 (Fig. 6f, g). Together, these data strongly indicate that *Sox2* expression in adult pancreatic epithelial cells is sufficient to drive alterations in lineage fate decisions, specifically initiating ADM and hyperproliferation, both early markers of pancreatic cancer.

## Discussion

Our data demonstrate the intricate interactions between chromatin, TF function, and transcription, specifically delineating how these forces work in concert to govern cell fate decisions from the earliest stages of gut organ specification and throughout development. We further report that disruption of one of these forces, namely TF expression, is fully sufficient to alter cell fate decisions, by either leading to a loss of identity in the intestine or completely changing cell fate in the pancreas.

Previously, bulk chromatin-profiling analyses showed both distinct and broadly similar chromatin patterns among digestive organs^20,21,50^. Notably, and for the first time in vivo, single-cell technology enabled us to analyze chromatin accessibility of the entire gut endoderm. We found that patterns of chromatin accessibility at E9.5 alone are sufficient to predict organ fate and future organ function. Moreover, our scATAC-seq data also revealed that the anterior-posterior orientation of organs is preserved in chromatin accessibility patterns, with organ identities projected onto a UMAP plot mirroring the spatial orientation of organs in vivo. Organ-specific patterns of chromatin accessibility also strongly overlap with organ-specific patterns of gene expression, indicating a relationship between chromatin accessibility, transcription, and organ identity occurs at the earliest stages of gut development, despite the morphological similarities along the primitive gut tube.

Moreover, the organ-specific relationships between TF activity in chromatin and transcription remain intimately linked over developmental time, with shifts in transcription strongly associated with changes in chromatin accessibility. These changes simultaneously reflect potential binding sites of TFs, such as HNF4α in the intestine, whose putative binding sites shift with chromatin accessibility over time to as organ specification occurs. From these analyses, we can hypothesize that TFs bind different open chromatin regions over time to regulate organ-specific patterns of gene expression, reinforcing lineage fate decisions over time from early gut morphogenesis to regionalization. However, we also observed that changes in key lineage-specific TF binding are sufficient to alter chromatin accessibility patterns and drive subsequent changes in lineage fate decisions. Embryonic deletion of *Sox2* led to a loss of chromatin accessibility patterns associated with differentiated gastric tissues, while embryonic deletion of *Cdx2* led to a shift in chromatin architecture from an intestinal pattern to a foregut pattern. In adult homeostasis, we demonstrate that *Sox2* expression is sufficient to drive loss of intestinal identity and ADM in pancreatic tissue, further supporting the powerful role of linage-specific TFs in regulating lineage fate decisions.

The interaction of chromatin accessibility and TF function in regulating lineage fate decisions of the gut is an active topic of exploration. While we demonstrate a strong association between dynamic chromatin accessibility patterns and organ-specific TF binding, most of our data do not provide sufficient evidence on causal relationships between them. Clearly, TFs have an effect on both chromatin accessibility and lineage fates^51^. While Forkhead box (FOX) and GATA families of TFs were demonstrated to be pioneering TFs required for liver induction^52^, it remains to be determined whether other organ-specific TFs in the gut may also possess pioneering activity during development. SOX2 is known to exhibit pioneering activity in embryonic stem cells and during reprogramming^53–55^, raising the possibility that gut organ-specific TFs such as SOX2 are the architects that direct chromatin accessibility patterns and subsequent lineage fate decisions in both development and disease. Increasing evidence suggests that pioneer factors mediate embryonic expression signatures in cancer^56^. Interestingly, a recent study has shown that when overexpressed, CDX2 could bind its adult intestinal sites that are inaccessible in the stomach, promoting intestinal metaplasia, a key early process in tumorigenesis^57^. Therefore, it will be important to further investigate pioneer TFs in gut development, stem cell homeostasis, and diseases such as cancer.

## Methods

### Experimental and model details

#### Mouse models

All mice used in this study were handled in accordance with the rules and regulations of the Canadian Council on Animal Care Guidelines for Use of Animals in Research and Laboratory Animal Care under protocols approved by the Animal Care Committee at The Centre for Phenogenomics (protocol: 19-0276H). Mice were subject to 12-h day/night cycles and were housed at 21–23 °C with an ambient humidity of 40–60%. *Shh*^*Cre*^ and *Pdx1*^*Cre/+*^;*R26*^*mTmG*^, and *Villin*^*CreERT2*^ mice were received from Dr. Chi-Chung Hui and Dr. Ramesh Shivdasani, respectively. *Mist1*^*CreERT2*^ mice were purchased from the Jackson Laboratory. *R26*^*Sox2-IRES-GFP*^ mice were previously described^58^.

Two sets of E9.5 embryos were used for scATAC-seq analysis. These mice were derived by breeding CD1 mice and harvesting all embryos, regardless of gender, as samples were pooled immediately to provide maximal numbers of fresh cells. Parental mice were housed together until dissection and were not subject to any treatment.

E17.5 (*n* = 3) and E9.5 (*n* = 3) *Pdx1*^*Cre/+*^;*R26*^*Sox2-IRES-GFP/mTmG*^ mice were used to examine *Sox2* overexpression in the intestine, pancreas, and stomach. These are mixed-strain mice, generated by crossing inbred *R26*^*Sox2-IRES-GFP/Sox2-IRES-GFP*^ mice to inbred *Pdx1*^*Cre/+*^;*R26*^*mTmG/mTmG*^ mice. To maximize the yield of mutant mice, all mutants were collected, regardless of gender. E17.5 (*n* = 3) and E9.5 (*n* = 3) *Pdx1*^*Cre/+*^;*R26*^*mTmG/+*^ mice used as lineage-traced controls were generated by crossing inbred *Pdx1*^*Cre/+*^;*R26*^*mTmG/mTmG*^ mice to CD1 mice. All parental mice used in these analyses we co-housed with their breeding partners until the scheduled dissection. No mice in these experiments were subject to any treatment.

E13.5 (*n* = 2) *Pdx1*^*Cre/+*^;*R26*^*mTmG/+*^ mice were used to isolate pancreatic epithelial cells for RNA-seq and ATAC-seq analyses. Parental mice were housed together until dissection and were not subject to any treatment. E13.5 (*n* = 2) *Shh*^*Cre/+*^;*R26*^*mTmG/+*^ mice were used to isolate lung epithelial cells for RNA-seq and ATAC-seq analyses. Parental mice were housed together until dissection and were not subject to any treatment.

*Villin*^*CreERT2/+*^;*R26*^*Sox2-IRES-GFP*^ (*n* = 4) and *Villin*^*+/+*^;*R26*^*Sox2-IRES-GFP/ +*^ (*n* = 3) littermate control mice were co-housed for the duration of the experiment. At 6 weeks of age, all mice were subject to tamoxifen treatment (20 mg/kg) through intraperitoneal (IP) injection for 3 days. Mice were then sacrificed 8 weeks after the last injection.

*Mist1*^*CreERT2/+*^;*R26*^*Sox2-IRES-GFP/+*^, *Mist1*^*+/+*^;*R26*^*Sox2-IRES-GFP/+*^, *Mist1*^*CreERT2/+*^;*R26*^*mTmG/+*^, and *Mist1*^*CreERT2/+*^;*R26*^*mTmG/+*^ mice all underwent the same tamoxifen regimen as *Villin*^*CreERT2/+*^;*R26*^*Sox2-IRES-GFP*^ and *Villin*^*+/+*^;*R26*^*Sox2-IRES-GFP/+*^ mice. *Mist1*^*CreERT2/+*^;*R26*^*Sox2-IRES-GFP/+*^ and *Mist1*^*+/+*^;*R26*^*Sox2-IRES-GFP/+*^ littermates were co-housed until dissection, as were *Mist1*^*CreERT2/+*^;*R26*^*mTmG/+*^ and *Mist1*^*+/+*^;*R26*^*mTmG/+*^ mice. *Mist1*^*CreERT2/+*^;*R26*^*Sox2-IRES-GFP/+*^ and *Mist1*^*+/+*^;*R26*^*Sox2-IRES-GFP/+*^ mice were sacrificed at two different time points: 1 day after tamoxifen treatment (*n* = 3 for both control and experimental mice) and 8 weeks after tamoxifen treatment (*n* = 4 for experimental mice and *n* = 3 for control mice). *Mist1*^*CreERT2/+*^;*R26*^*mTmG/+*^ and *Mist1*^*+/+*^;*R26*^*mTmG/+*^ mice were only sacrificed 1 day after tamoxifen treatment (*n* = 3 for both models).

### Method details

#### Single-cell ATAC-seq sample preparation

For each biological replicate (*n* = 2), E9.5 gut tubes were micro-dissected, washed in cold PBS and digested in 4 ml of a 2:1 solution of Trypsin LE: 1× PBS at 37 ˚C. After 10 minutes, tissue was vigorously pipetted, until the organs broke down into a single-cell suspension, which was confirmed using brightfield microscopy. The enzymatic reaction was then inhibited with 10% FBS. After washing cells in cold PBS, the single cells were re-suspended in 400 µl of 1:5000 Sytox Blue (ThermoFisher Scientific, S34857) and 1:200 EPCAM (Abcam, ab95641) in cold 1× PBS, filtered through a 30 µm mesh and submitted for sorting. Live EPCAM+ cells were sorted for subsequent nuclear isolation and preparation with the Chromium Single-Cell ATAC Library Kit (10× Genomics, PN-1000111) as per the manufacturer’s guidelines. Sequencing was performed on the Illumina HiSeq2500 using paired-end 50 base-pair reads.

#### Bulk RNA-seq preparation

Two biological replicates (*n* = 2) at E13.5 were used for bulk RNA-seq experiments. *Shh*^*Cre/+*^;*R26*^*mTmG/+*^ mice were used to isolate lung epithelial cells, while *Pdx1*^*Cre/+*^;*R26*^*mTmG/+*^ mice were used to isolate pancreatic epithelial cells. Tissues were prepared for sorting in the same manner as E9.5 samples but did not undergo EPCAM staining. Live, GFP-expressing cells were sorted and used for RNA isolation using the RNAeasy Micro Plus kit (Qiagen, 74034). RNA libraries were prepared using the NEB Ultra II Directional RNA Library Prep Kit (New England Biolabs, E7765). SIRV-Set 3 synthetic RNA spike-in was used at the manufacturer’s recommendations to help normalize batch effects (Lexogen, 051.01) Samples underwent paired-end sequencing (2 × 125 base pair) on the Illumina HiSeq 2500 platform.

#### Bulk ATAC-seq preparation

Two biological replicates (*n* = 2) at E13.5 were used for bulk RNA-seq experiments. *Shh*^*Cre/+*^;*R26*^*mTmG/+*^ mice were used to isolate lung epithelial cells, while *Pdx1*^*Cre/+*^;*R26*^*mTmG/+*^ mice were used to isolate pancreatic epithelial cells. Tissues were prepared for sorting in the same manner as E9.5 samples but did not undergo EPCAM staining Live, GFP-expressing cells were sorted and prepared using the omni-ATAC-seq nuclear isolation protocol^59^. Samples underwent paired-end sequencing (2 × 125 base pair) on the Illumina HiSeq 2500 platform.

#### Whole-mount imaging

Whole-mount images of embryonic and adult samples were taken after samples were fixed in 4% PFA overnight, washed for 30 min in PBS, and stored in 70% ethanol. The *Mist1*^*CreERT2/+*^;*R26*^*Sox2-IRES-GFP/+*^ whole-mount image was generated by taking 2 images and stitching the images together using the Pairwise Stitching function in ImageJ.

#### Immunohistochemistry and histology

Tissue samples used in immunohistochemistry and histology analyses were fixed in 4% PFA at 4 °C overnight. E9.5 samples were washed in PBS and placed in 30% sucrose for 48 h. Samples were washed in PBS set in OCT for 1 h before being frozen on dry-ice and cut into 5 µm sections. All remaining tissues were washed in PBS and placed in 70% ethanol before processing and embedding in paraffin wax. Paraffin-embedded samples were sectioned at 5 µm.

Samples used in histology were rehydrated and stained with Harris’ Hematoxylin (Electron Microscopy Sciences, 26041-06) and Eosin Y (Electron Microscopy Sciences, 26051-11).

For immunofluorescence analyses, frozen sections were rehydrated in PBS and subject to antigen-retrieval (10 mM Tris-EDTA, pH 8.0), while paraffin sections were deparaffinized and rehydrated before undergoing the sample antigen-retrieval process. All sections were blocked with 10% goat serum in PBST (0.5% TWEEN-20, Millipore Sigma, 11332465001) and incubated with primary antibodies at 4 °C overnight in 10% goat serum in PBST. Samples were washed in PBST and incubated with secondary antibodies and nuclear Hoechst stain (Cell Signaling Technology, 4082 S) in the dark for 1 h at room temperature before mounting and imaging. Primary antibodies used here are as follows: SOX2 (Abcam, ab97959, 1:200), GFP (Abcam, ab13970, 1:1000), OLFM4 (Cell Signaling Technology, 39141S, 1:300), CDX2 (Cell Signaling Technology, D11D10, 1:200), Lysozyme C (DAKO, A0099, 1:1000), PDX1 (DSHB F109-D12, 1:200), Amylase (Abcam, ab21156, 1:500), MUC1 (Invitrogen, MA5-11202, 1:300), Ki67 (Abcam, ab15580, 1:500), TFF3 (Gift from D. Podolsky, University of Texas Southwestern, Dallas, TX, 1:500). Secondary antibodies used are as follows: Alexa Fluor 568 (Invitrogen, A11011, 1:500), Alexa Fluor 488 (Invitrogen, A11029, 1:500), Alexa Fluor 488 (Invitrogen, A11039, 1:500), Alexa Fluor 568 (Fisher Scientific, 127585160, 1:500).

#### Quantification and statistical analysis

To assess proliferation as marked by Ki67+ cells, we took 3 images at ×20 magnification for each sample. We counted the number of DAPI+ cells and the number of Ki67 per field of view, calculating a percentage of proliferating cells per field of view. The average of these three fields was taken to be a single biological replicate. Unpaired, two-tailed T-tests with Welch’s Correction were used to statistically assess the differences between the mean of *n* = 3 (±SEM) biological replicates per experimental group.

#### Bulk data processing and analysis

Bulk data processing: Bulk RNA-seq data were aligned to the mouse reference genome mm10 using HISAT2^60^ with default parameters. Mapped reads were further filtered by MAPQ (> = 30) using SAMTOOLS^61^, and chimeric reads were discarded. The gene expression index was summarized using HTSEQ (count)^62^ and StringTie (rpkm)^63^.

The publicly available microarrays of embryonic *Cdx2* knockouts (Agilent Technology) were downloaded from the European Bioinformatics Institute (EBI) ArrayExpress (accession numbers E-MTAB-92)^12^. The *gMeanSignals* were extracted from raw data and regarded as the gene expression index after quantile normalization. R limma package^64^ was then used to detect differentially expressed genes between the KO ileum samples and controls with BH-adjusted *P*-value <0.05 and fold change >1.5 as the threshold.

Bulk ATAC-seq data were aligned to the mouse reference genome mm10 using Bowtie2^65^ (with additional parameters ‘-X 2000 -3 76’). Mapped reads were further filtered by MAPQ (> = 30), and chimeric reads were discarded. Bulk ATAC-seq peaks were detected using MACS2^66^ (with additional parameters ‘-q 0.01 --keep-dup all --nomodel --extsize 100’).

ChIP-seq data were aligned to the mouse reference genome mm10 using Bowtie2 with default parameters. Mapped reads were further filtered by MAPQ (> = 30). ChIP-seq peaks were detected using MACS1.4 (with additional parameters ‘-p 1e-5 --keep-dup 1 --nomodel --shiftsize 73’). Peak co-occurrence between any two peak sets (e.g., ATAC-seq peaks) was determined by 1 bp overlapping between peak regions.

Identification of organ-specific genes and ATAC-seq peaks: Organ-specific genes and ATAC-seq peaks from E13.5 data were identified by K-means clustering. For organ-specific genes, we first performed quantile normalization followed by a log transformation on the expression index matrix (FPKM) to get a log-transformed matrix of normalized expression. Next, 2000 genes with highest index of dispersion (variance/mean log2(norm.Exp), detected from E13.5 bulk RNA-seq data across organs) were selected as high variance genes. K-means clustering was then performed on the high variance genes’ expression matrix. The organ-specific clusters were presented by heatmap.

For organ-specific ATAC-seq peaks, MACS2 narrow peaks from each of all bulk ATAC-seq samples at the same stage were first merged to a merged peak set. Next, the sequencing depth-normalized read count was calculated on each of the merged peak for each sample and combined as an accessibility count matrix. Then K-means clustering was performed after quantile normalization and log transformation of the accessibility count matrix. Peak clusters in which only one organ has high accessibility were selected as candidate organ-specific clusters. Within a candidate organ-specific cluster, peaks that have a significantly higher signal in the target organ than other organs (fold change > = 2.5 in the target organ compared to the average of the other organs) were selected as organ-specific ATAC-seq peaks and used for the following analyses.

Identification of gained and lost peaks after knock-out experiments: The sequencing depth-normalized read counts were calculated on the merged peak set from both knock-out (KO) and wild-type (WT) samples. The signal fold change between KO and WT was calculated for each merged peak. Peaks with a fold change greater than 2 were identified as KO-gained or KO-lost peaks. All remaining peaks were identified as maintained peaks.

Pre-defined *Cdx2*-KO differentially expressed genes and WT differentially expressed genes were categorized by their peak association. Peak-associated genes are defined as genes whose flanking region (up/downstream 50 kb from the transcriptional start site, TSS) contains the target peak. UpSet plots were generated using R package UpSetR^67^. The functional annotation of genes associated with different peak sets was performed using DAVID^68,69^.

E9.5 to E13.5 lineage restriction analysis: To get E9.5 foregut common peaks, we first merged all the peaks from the four foregut pseudo-bulk organs, esophagus, pharynx, stomach, and lung using the iterative-overlap method through ArchR function *addReproduciblePeakSet* to produce a E9.5 foregut peak set. Then, overlaps between the E9.5 foregut peak set and peaks from each of the foregut pseudo-bulk organ were identified. The E9.5 foregut peaks overlapped with as least one peak from each of the foregut organs were retained as E9.5 foregut common peaks. E9.5 foregut common peaks were then compared with ATAC-seq peaks from E13.5 stomach and E13.5 lung, respectively. In this way, peaks that are commonly open at E9.5 but closed in either organ at E13.5 were identified.

We next calculated a fold change (pseudo count = 0.5) between the log-transformed normalized expression at E13.5 and that at E9.5 for each gene. Genes with fold change less than 0.5 were defined as genes repressed during the development from E9.5 to E13.5. In this way, we successfully identified a repressed gene list for stomach and lung, respectively. By comparing the two repressed gene lists, genes were categorized as genes repressed in both organs, genes specifically repressed in stomach, and genes specifically repressed in lung. Each gene category was then refined with the corresponding closed peak set to research the repressed genes accompanied with the close of chromatin regions nearby. For example, genes specifically repressed in stomach were refined with peaks specifically closed in stomach, so that only the genes associated with any closed peak were retained as stomach-specific repressed genes accompanied with closed peaks. Peak-associated genes are defined as genes whose flanking region (up/downstream 50 kb from the transcriptional start site, TSS) contains the target peak. GO-term analyses were then performed on each refined gene list using DAVID^68,69^.

#### Single-cell sequencing data processing and analysis

scRNA-seq processing: A public scRNA-seq dataset (GSE136689)^10^ of mouse foregut at E9.5 was utilized in our research. The expression matrix (count matrix) and meta file were downloaded for following analysis. Single-cell data expression matrix was processed with the R package Seurat (version 3.0)^22^. The expression data was first normalized using function “NormalizeData” with “LogNormalize” method. Highly variable genes were then identified by function “FindVariableFeatures” with default parameters. After principal component analysis (PCA), the JackStraw-based resampling test was applied to determine the number of dimensions retained. At the reduced dimensionality, UMAP embeddings were generated for visualization and cells were labeled using the existing annotation in the meta file.

scATAC-seq processing: scATAC-seq data were pre-processed using the cellranger-atac package (genome version mm10). The ArchR package^70^ was used for quality control (QC) and basic analysis. In detail, cells with <1000 ATAC-seq fragments, TSS enrichment score less than 4, or doublets were discarded. The two scATAC-seq samples were combined for the subsequent analysis. Dimensionality reduction and cell clustering were then performed using ArchR with default parameters. For data visualization, UMAP embedding with Harmony-adjusted^71^ LSI dimensions were calculated and plotted as a scatter plot with cells color-coded according to specific features (described below).

Cell type labeling: For organ label assignment to cell clusters, a “gene score” was calculated for each gene in each individual cell using ArchR^70^ under default parameters. In brief, the “gene score” for each gene in each cell was calculated by summarizing scATAC-seq signals in all 500 bp tilling bins within 100 kb from the gene locus, weighted by the distance from each bin to the gene and normalized considering the gene length and the total sequence read count in the cell. The distance weight was estimated for each gene-bin pair based on the distance between them. The gene length scale factor was estimated based on the total region size for each gene (gene length and 100 kb flanking regions on both sides) and scaled for 5 levels.

Marker genes of different organs were collected from previous publications^12–14,16,19,23–26,72–77^. UMAP plots colored by the gene scores of marker genes were generated for visualization and the organ label was assigned to clusters based on the gene score distribution of the marker genes of that organ. Particularly, cluster 8 (Figure S1c) was regarded as combination of the stomach, lung, and esophagus, and cluster 9 was mainly composed of esophagus cells. To distinguish between stomach, lung, and esophagus, we performed a sub-clustering using the cells from both clusters 8 and 9, followed by the marker gene labeling. As a result, subcluster 1 (Figure S1d) was identified as stomach and renamed as cluster 8a in the original dataset, while subcluster 4 was identified as lung and renamed as cluster 8b in the original dataset. All other subclusters were identified as esophagus and merged into cluster 9 in the original dataset.

To further validate the cell type identification, we used the Seurat “Reference Assembly Integration” method^22^ built in the ArchR package’s unconstrained integration workflow^70^ to perform integration analysis of a scRNA-seq dataset (GSE136689)^10^ with the scATAC-seq dataset and to transfer cell labels from scRNA-seq to scATAC-seq (Fig. 1d). In detail, to integrate two single-cell datasets with shared biological states, Seurat first applied canonical correlation analysis (CCA) for dimensionality reduction on both datasets. At the reduced-dimensional representation, Seurat used mutual nearest neighbors (MNN) to identify cell pairs (one from scATAC-seq and the other from scRNA-seq) as “anchors” connecting the two datasets. Next, Seurat scored the anchors based on the consistency of the cell pair in the cluster structure of each dataset and associated each cell from scATAC-seq to each anchor with a weight matrix considering the cell-to-anchor distance and the anchor score. Finally, Seurat integrated the cell label information from scRNA-seq for each anchor with the cell-to-anchor weight matrix to calculate a label prediction score for each cell from scATAC-seq, and subsequently transferred the cell label information from scRNA-seq to scATAC-seq. For each cell in the scATAC-seq dataset, the organ label from scRNA-seq with the highest prediction score was assigned as the transferred cell label of this cell in scATAC-seq. The pre-identified colon cells and other unidentified cells were colored in gray because the colon cells were unidentified in the scRNA-seq dataset.

Downstream analysis: After organ labeling, scATAC-seq reads from the same organ were merged as a pseudo-bulk ATAC-seq dataset and underwent reproducible peak calling using ArchR. The reproducible merged peak set was produced using an iterative overlap peak merging strategy implemented in ArchR. Organ-specific genes and organ-specific peaks were also detected using ArchR with parameters “FDR < = 0.05 & log2(fold change) > = 1.5” and “FDR < = 0.05 & log2(fold change) > = 1”, respectively. The functional annotation of organ-specific peaks was performed using GREAT^78^. Top 10 Gene Ontology Biological Processed (BP) terms with smallest adjusted *P*-values for each organ were plotted in Fig. 1c. Motifs enrichment analysis was performed using MDSeqPos^79^ (with additional parameters ‘ -w 600 -p 0.001’) for organ-specific peaks. Peak enrichment analysis of organ-specific peaks was performed using ArchR’s peakAnnoEnrichment function with parameters “FDR < = 0.1 & log2(fold change) > = 1”, and the -log10 adjusted *p*-values for the analysis were plotted as heatmap (Fig. 2b). To map the peaks from each category onto the scATAC-seq data from E9.5 and quantify the enrichment of each peak category in each cell, we applied the chromVAR method^39^ integrated in the ArchR package^70^ using the peak set from each category as a custom region set. For an input peak set, chromVAR computes a “deviation score” for each cell to quantify the relative chromatin accessibility level of these peaks in this cell compared to the average accessibility of these peaks in all cells, representing the degree of peak enrichment on the single-cell level (Fig. 3e, Figure S9B). The same peak calling solution (macs2, ‘-q 0.01 --keep-dup all --nomodel --extsize 100’) was applied to both bulk ATAC-seq data and pseudo bulk ATAC-seq data generated from scATAC-seq reads for a fair comparison.

#### Temporal analysis

Association between chromatin accessibility and gene expression: To explore the organ-specific/marker genes’ regulatory function, we first selected top 200 upregulated genes and top 2000 accessibility-increased peaks with the greatest fold change for each organ and compared their genome-wide distribution using an “association score”. The “association score” measured the association between a given gene set and a peak set. We defined the Association score:

1 $${{{{{\rm{Association}}}}}}\,{{{{{\rm{score}}}}}}=\frac{{O}_{{{{{{\rm{T}}}}}}}/{G}_{{{{{\rm{T}}}}}}}{O/G}$$

Where *G* is the total number of genes (mm10, refseq annotation), and *O* is the number of genes whose flanking region (up/downstream 50 kb from the transcriptional start site, TSS) contains at least a target peak. *G*_T_ is the number of target genes, which is 200 in this case, and *O*_T_ is the number of target genes whose flanking region (up/downstream 50 kb from the transcriptional start site, TSS) contains at least a target peak. The higher the “association score” is, the greater association the gene set and the peak set have.

Relative motif enrichment score: We defined a “relative motif enrichment score” to measure the relative TF motif enrichment between two developmental stages (e.g., E9.5 vs. E13.5). The “relative motif enrichment score” for a given organ was calculated as follows:

1. The ATAC-seq signal (normalized read count) at both stages was calculated on the merged peak set, and signal fold change between the two stages was calculated for each peak.

2. We collected motifs of 353 mouse TFs from HOCOMOCO^80^ database. Then, the genome-wide motif sites for each TF were identified using FIMO^81^ in the MEME suite^82^ across mouse reference genome mm10.

3. The merged peaks were grouped according to a given TF’s motif occurrence on the peaks (motif-overlapping group or motif-nonoverlapping group).

4. A two-sided Wilcoxon test was performed on the ATAC-seq signal fold changes comparing the two peak groups. The “relative motif enrichment score” was defined as the -log10 p-value of the test statistic if the motif-overlapping group has higher fold changes, or log10 *p*-value of the test statistic if the motif-overlapping group has lower fold changes. In this way, a positive relative motif enrichment score indicated increased accessibility during development, and a negative relative motif enrichment score indicated decreased accessibility.

For the motif analyses between E9.5 and E13.5, we highlighted the top 20 TFs for each timepoint (Fig. 2e). For the motif analyses between E13.5 and E16.5, top 5% TFs for each timepoint on either X-axis or Y-axis are highlighted (Fig. 2f).

### Reporting summary

Further information on research design is available in the Nature Research Reporting Summary linked to this article.

## Supplementary information

Supplementary Information

Description of additional Supplementary File

Supplementary Dataset 1

Supplementary Dataset 2

Supplementary Dataset 3

Supplementary Dataset 4

Supplementary Dataset 5

Supplementary Dataset 6

Reporting Summary

## Data Availability

scATAC-seq, bulk ATAC-seq and RNA-seq data generated in this study have been deposited in the NCBI Gene Expression Omnibus under accession code “GSE168373”. The data of E9.5 scRNA-seq, E13.5 and E16.5 intestine and stomach ATAC-seq, E16.5 RNA-seq, E16.5 intestinal epithelium ATAC-seq, CDX2 ChIP-seq, and SOX2 ChIP-seq used in this study are available in the NCBI Gene Expression Omnibus under accession code “GSE136689”, “GSE134275”, “GSE134276”, “GSE115314”, and “GSE134582” respectively. Microarray data of E18.5 Cdx2-KO ileum and controls used in this study are available in the European Bioinformatics Institute (EBI) ArrayExpress under accession code “E-MTAB-92”. Mouse lines generated by this project are available upon request, however, these are subject to transportation restrictions and may require a material transfer agreement to acquire. No other unique reagents were generated by this study. All genomics datasets used in this study are summarized in Supplementary Dataset 6. All other relevant data supporting the key findings of this study are available within the article and its Supplementary Information files or from the corresponding author upon reasonable request. Source data are provided with this paper.

## Source data

Source Data

## Code availability

All computational code and scripts are available on GitHub at https://github.com/zang-lab/Single-cell-chromatin-profiling-of-the-primitive-gut-tube and Zenodo at 10.5281/zenodo.6394842^83^.
