# Supplementary material for: Single-cell chromatin profiling of the primitive gut tube reveals regulatory dynamics underlying lineage fate decisions
Source: Nat Commun. 2022 May 26;13:2965. doi: 10.1038/s41467-022-30624-w (PMC9135761; doi:10.1038/s41467-022-30624-w)
Supplement: Supplementary file 1 — Supplementary Information [file 41467_2022_30624_MOESM1_ESM.pdf]

Supplementary Information

Single-cell chromatin profiling of the primitive gut tube reveals regulatory dynamics underlying lineage fate decisions

Ryan J. Smith[1,2,*], Hongpan Zhang[3,4*], Shengen Shawn Hu[3*], Theodora Yung[1,2], Roshane Francis[1,2], Lilian Lee[1], Mark W. Onaitis[5], Peter B. Dirks[1,2], Chongzhi Zang[3,4,6‡], Tae-Hee Kim[1,2,‡]

[1]Program in Developmental & Stem Cell Biology, The Hospital for Sick Children, Toronto, Ontario M5G 0A4, Canada; [2]Department of Molecular Genetics, University of Toronto, Toronto, Ontario M5S 1A8, Canada; [3]Center for Public Health Genomics, University of Virginia School of Medicine, Charlottesville, VA, USA; [4]Department of Biochemistry and Molecular Genetics, University of Virginia, Charlottesville, VA, USA; [5]Division of Cardiovascular and Thoracic Surgery, University of California San Diego Medical Center, San Diego, CA, USA; [6]Department of Public Health Sciences, University of Virginia, Charlottesville, VA, USA

* These authors contributed equally.

Keywords: Gut endoderm; chromatin; transcription factors; organogenesis

[‡]**Corresponding authors:**

Tae-Hee Kim, PhD (corresponding author during review) **OR** Chongzhi Zang, PhD

The Hospital for Sick Children	University of Virginia
Toronto, ON M5G 0A4	Charlottesville, VA 22908, USA
Email: tae-hee.kim@sickkids.ca	zang@virginia.edu
Tel.: +1 (416) 813-8138	+1 (434) 243-5397

This file includes Supplementary Figures 1 to 12 and Supplementary Table (1 to 6) Legends.

Supplementary Figure 1

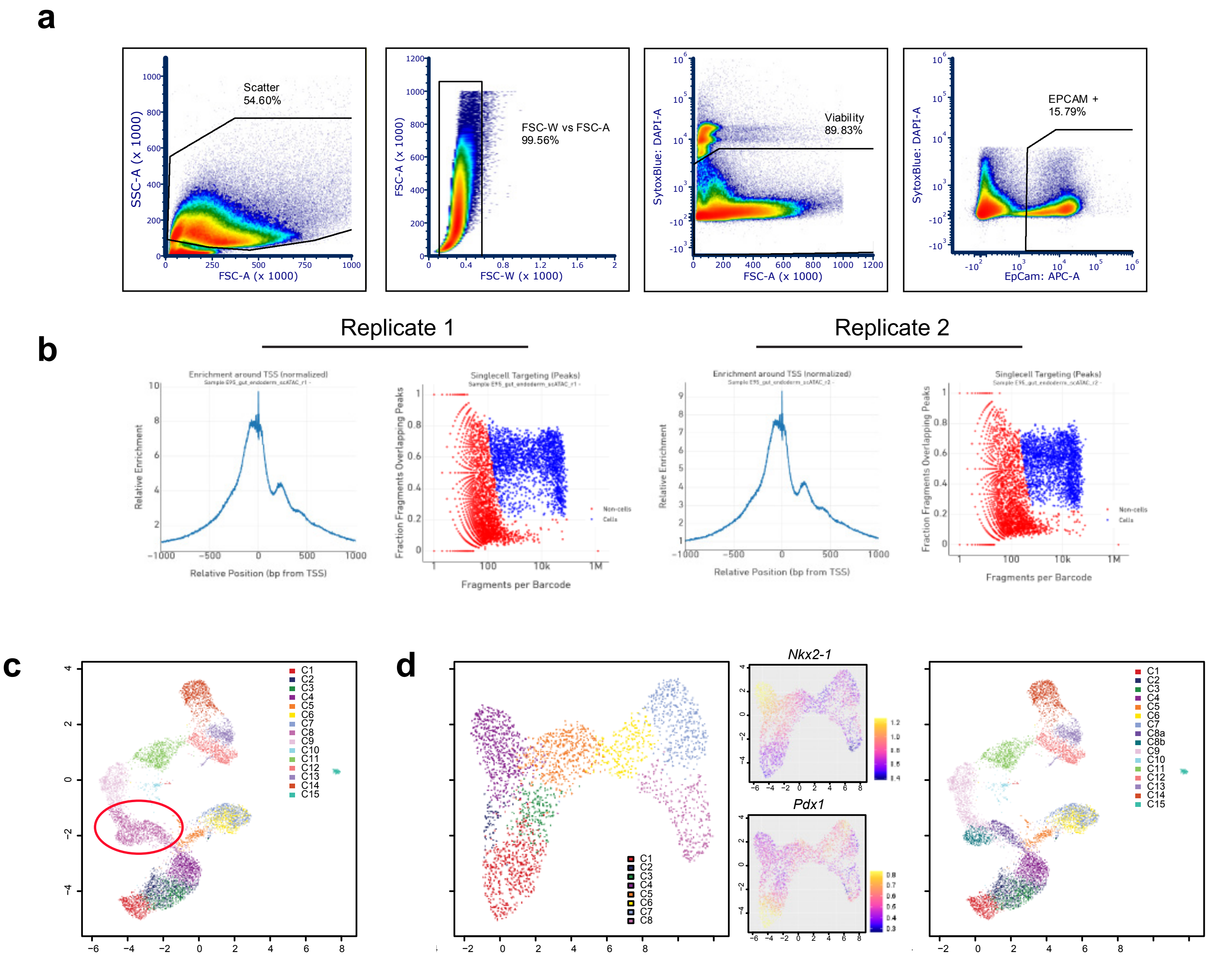

Figure S1. scATAC-seq on isolated endodermal cells reveals organ-specific patterns of chromatin accessibility concordant with transcriptional profiles. (A) Example of fluorescence activated cell sorting gating strategy used to isolate EPCAM-expressing cells of the E9.5 gut tube. (B) Quality control analyses for E9.5 scATAC-seq replicates: normalized enrichment at TSS (left), and single-cell targeting (right). (C) UMAP demonstrating the clustering of cells. Red oval marks cluster 8 for sub-clustering. (D) Process of sub-clustering cluster 8 by promoter accessibility at Nkx2-1 and Pdx1 to identify stomach (cluster 8a) and lung (cluster 8b).

Supplementary Figure 2

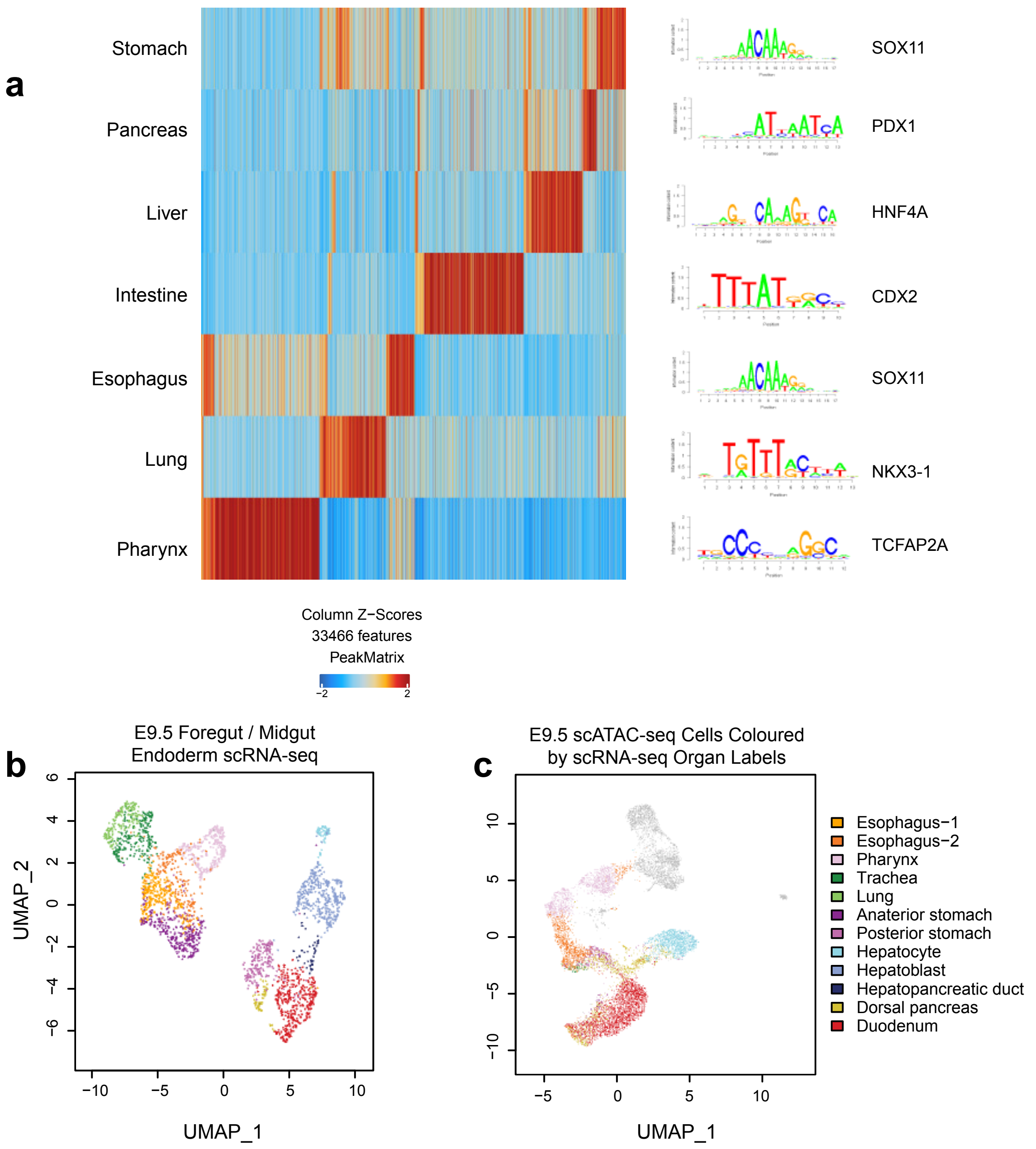

Figure S2. Integration of scRNA-seq and scATAC-seq in the primitive gut tube. (A) E9.5 scATAC-seq peaks clustered by organ label (left), with an example of an enriched transcription factor motif (right). (B) E9.5 gut endodermal scRNA-seq with cell types labeled. (C) E9.5 gut endodermal scATAC-seq labeled by scRNA-seq cell labels.

Supplementary Figure 3

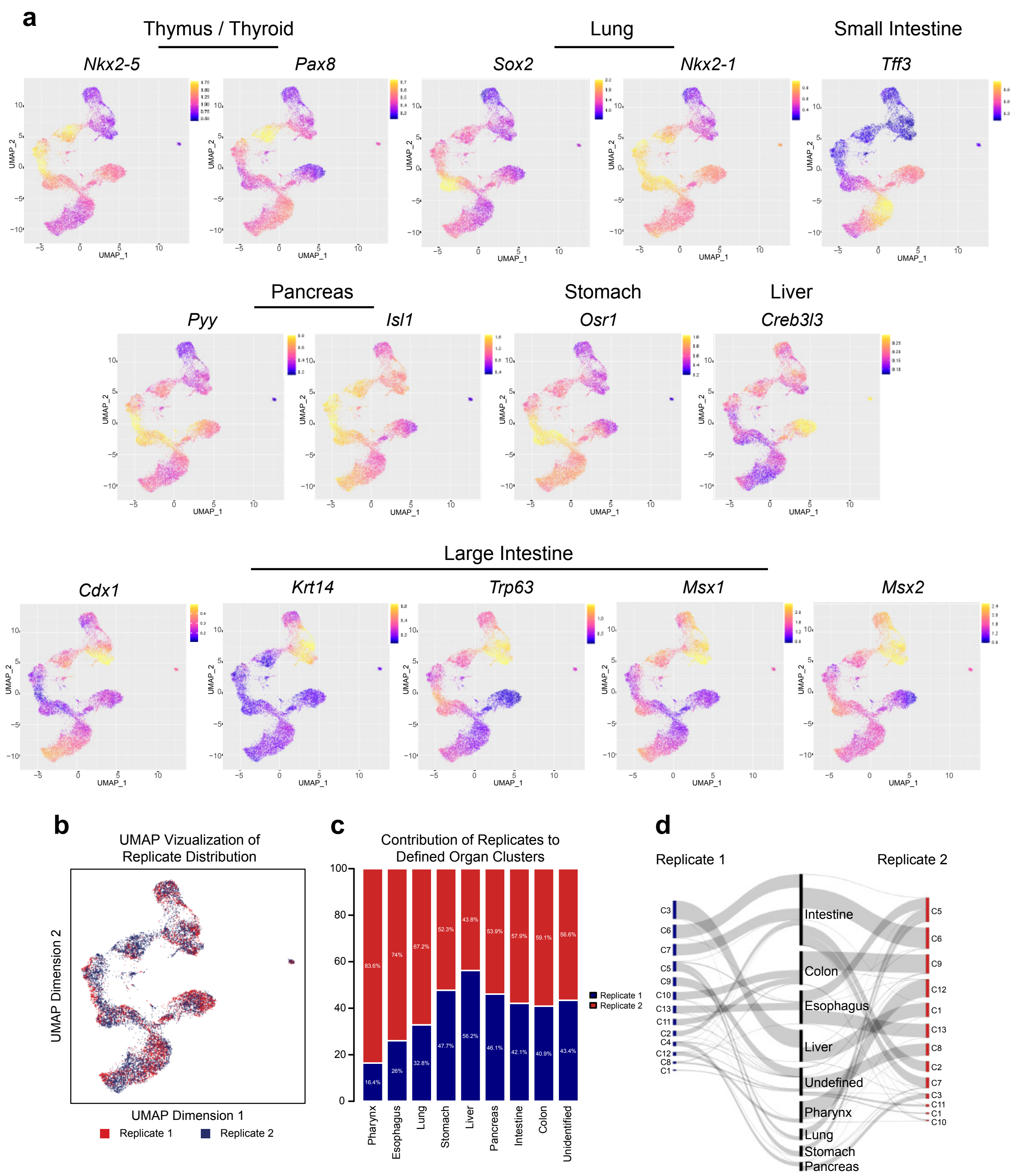

Figure S3. Assignment of organ identity. (A) UMAP scatter plots colored by gene score of known lineage-associated markers. In conjunction with those in Figure 1E, these plots are used to assign organ labels to individual cells. (B) UMAP visualization of scATAC-seq replicate distribution. (C) Contribution of each replicate to organ clusters assigned in Figure 1B (scRNA-seq, *N* = 12067 cells). (D) Sankey diagram demonstrating how cells from unassigned clusters from each replicate contribute to assigned organ clusters in Figure 1B.

Supplementary Figure 4

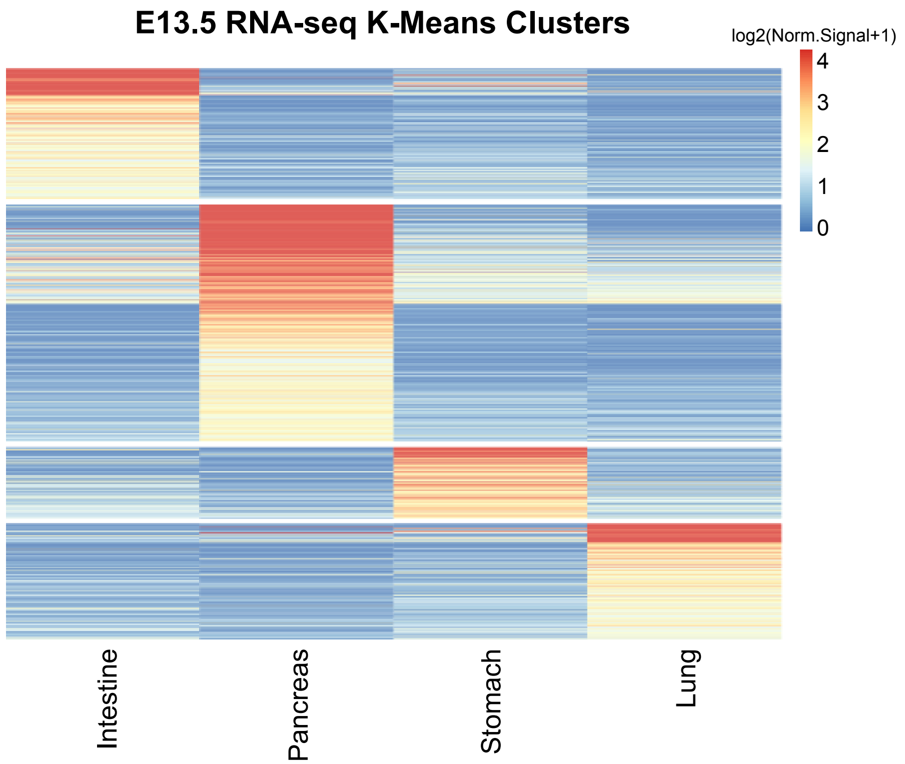

Figure S4. Tissue-specific patterns of gene expression at E13.5. Expression pattern of E13.5 organ-specific genes in the intestine, pancreas, stomach, and lung. Genes are ordered by K-means clustering of expression in the 4 organs. Color scale represents log-transferred normalized gene expression.

Supplementary Figure 5

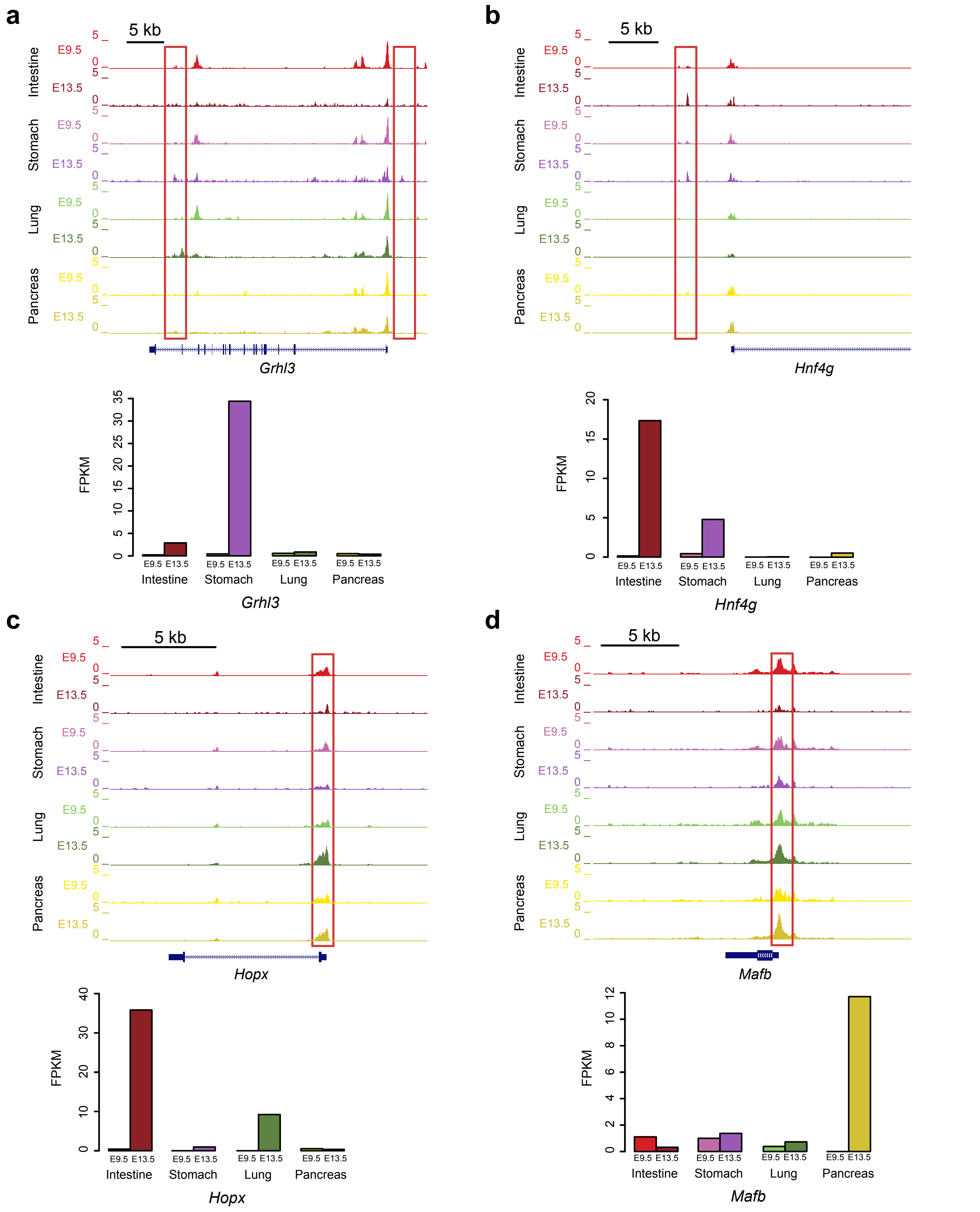

Figure S5. Temporal dynamics of chromatin accessibility and gene expression at marker genes across tissues. Chromatin accessibility and gene expression at example genes (A: Grhl3, B: Hnf4g, C: Hopx, D: MafB). Top: genome browser snapshot of ATAC-seq signal at E9.5 and E13.5 at marker genes in the intestine, stomach, lung, and pancreas. Bottom: gene expression (FPKM) for each marker gene at E9.5 (scRNA-seq, *N* = 1153 cells) and E13.5 (bulk, *N* = 8 samples) in the intestine, stomach, lung, and pancreas.

Supplementary Figure 6

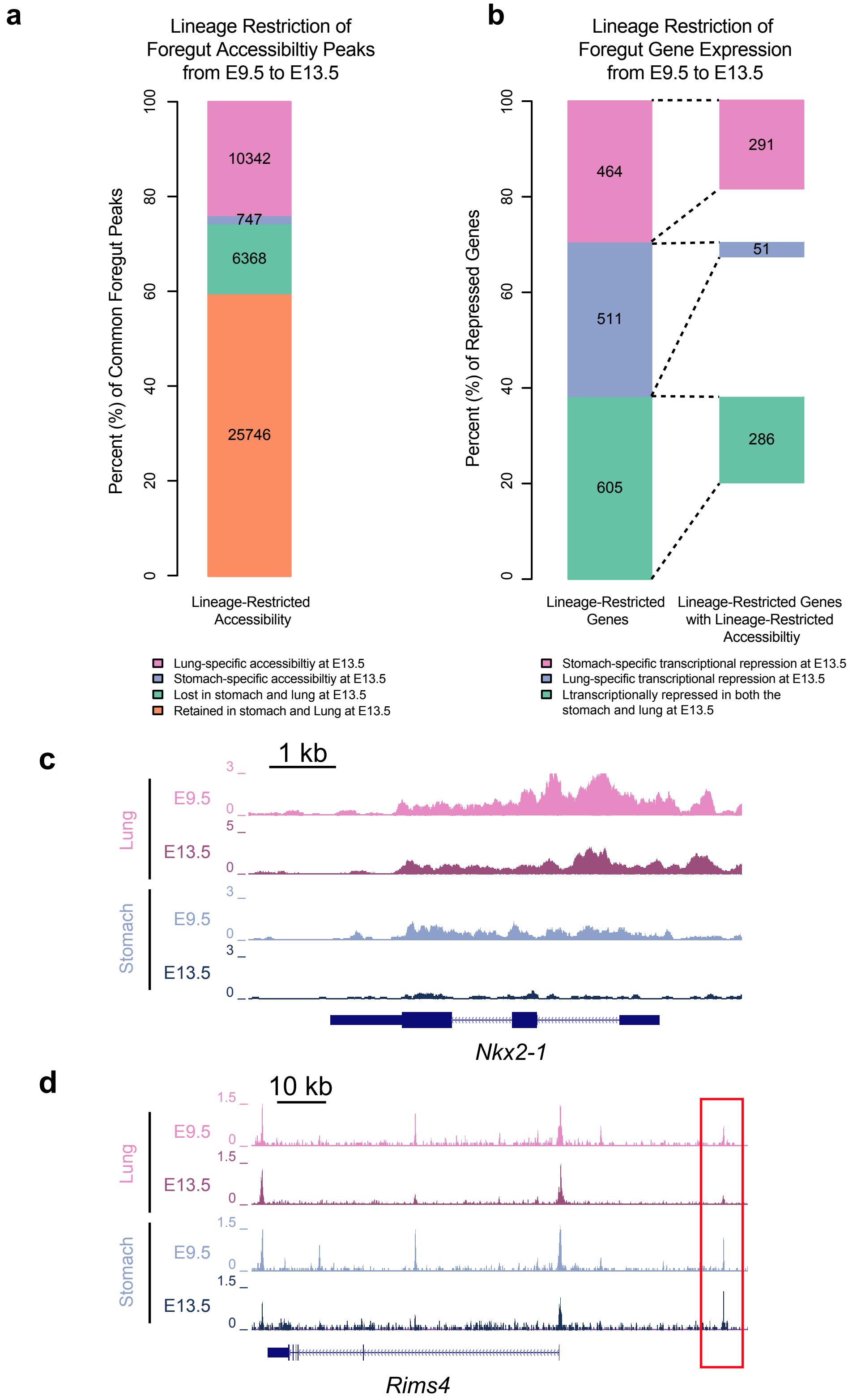

Figure S6. Lineage restriction of gene expression and chromatin accessibility in foregut development. (A) Composition of ATAC-seq peaks that are common in the E9.5 foregut (stomach, lung, esophagus, pharynx). Peaks are categorized based on their retaining in lung and stomach at E13.5. The numbers of peaks in each category are labeled on the chart. (B) Composition of lineage-restricted genes that are common in the E9.5 stomach and lung and change at E13.5. Left chart shows the total numbers of genes that are repressed in the lung, stomach or both organs from E9.5 to E13.5. Right chart shows the number of genes in each category that also show the same lineage-restriction pattern in chromatin accessibility from E9.5 to E13.5. (C) Genome browser snapshots (left) showing chromatin accessibility and bar plots (right) showing expression (FPKM) at Nkx2-1 locus, a lung-specific gene at E9.5 and E13.5 in the lung and stomach endoderm. (D) Genome browser snapshots (left) showing chromatin accessibility and bar plots (right) showing expression (FPKM) at Rims4 locus, a stomach-specific gene at E9.5 and E13.5 in the lung and stomach endoderm.

Supplementary Figure 7

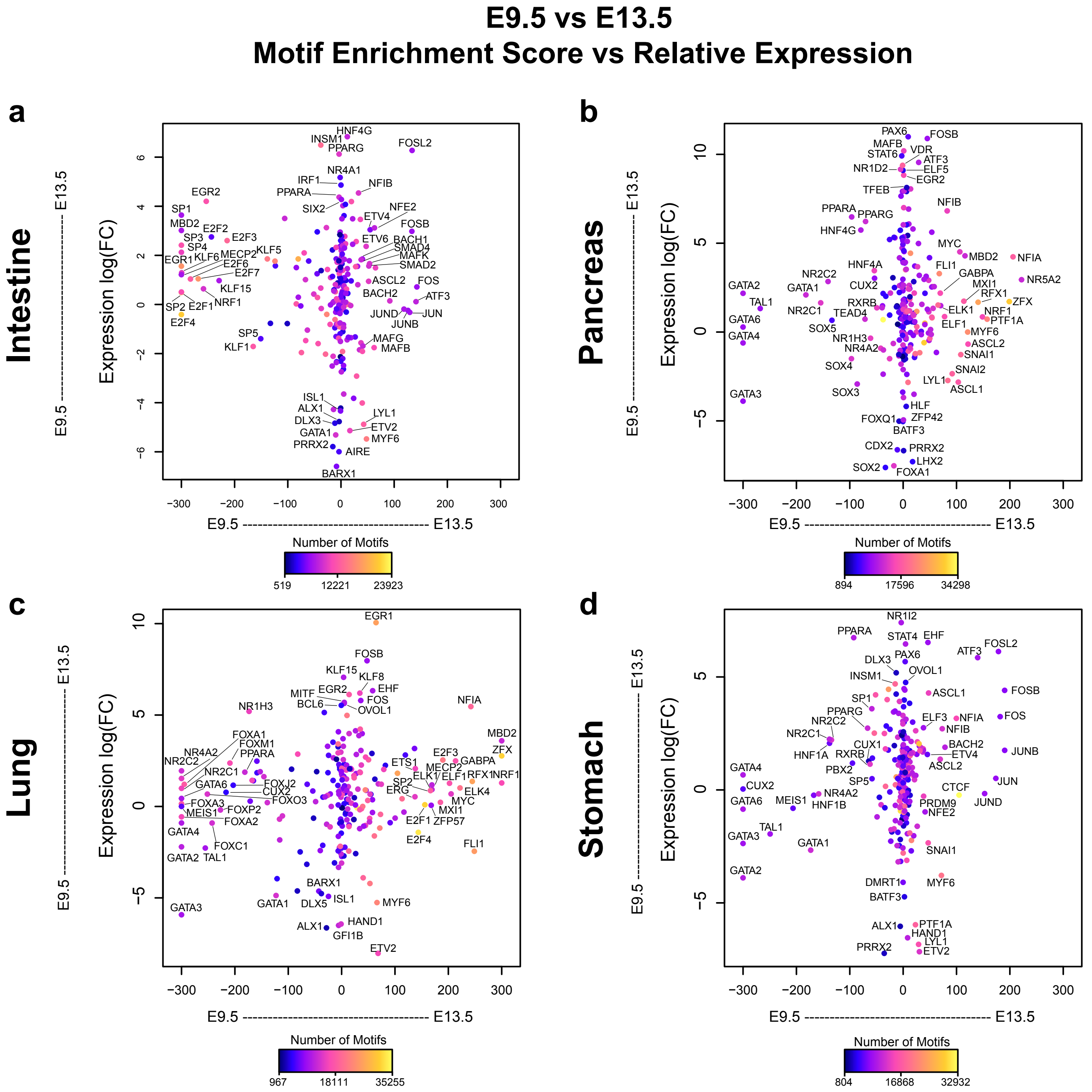

Figure S7. Relationship between transcription factor binding site accessibility and expression during organogenesis. DNA sequence motif enrichment in the open chromatin regions, ranked by relative motif enrichment scores (x-axes, see Methods for details) in intestine (A), pancreas (B), lung (C), and stomach (D) between E9.5 (scRNA-seq, *N* = 1153 cells) and E13.5 (*N* = 7 samples). Y-axes indicates the expression log fold change of these TFs. Top 20 TFs on each side of x-axes and top 10 TFs on each side of y-axes are labeled. Each data point is colored based on the total number of hits of this TF's motifs in the open chromatin regions.

Supplementary Figure 8

E13.5 vs E16.5
Motif Enrichment Score vs Relative Expression

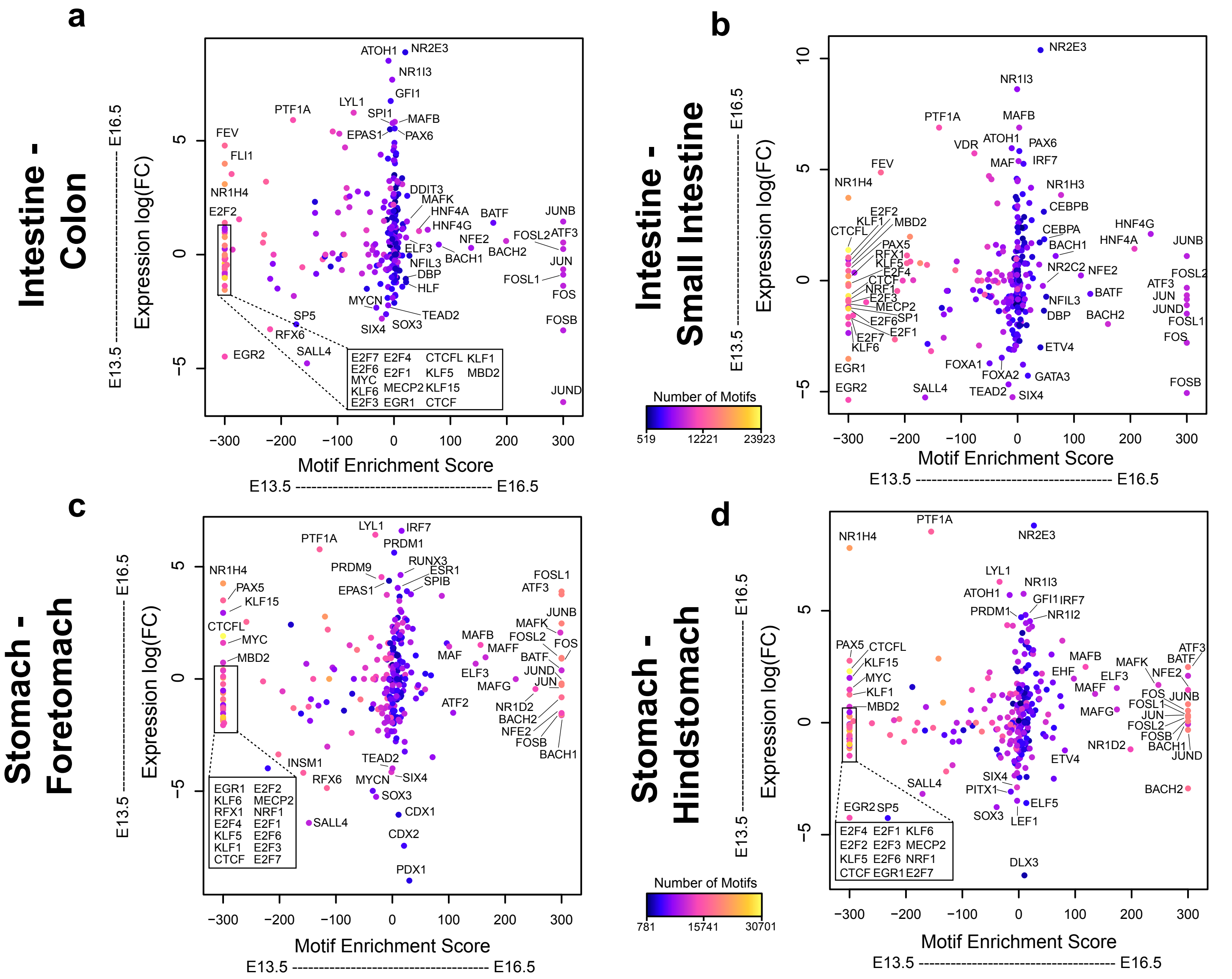

Figure S8. Relationship between transcription factor binding site accessibility and expression during regionalization. (A-B) DNA sequence motif enrichment in the open chromatin regions, ranked by relative motif enrichment scores (x-axes, see Methods for details) in between E13.5 intestine and E16.5 colon (A) or small intestine (B). *N* = 3. Y-axes indicates the expression log fold change of these TFs. Top 20 TFs on each side of x-axes and top 10 TFs on each side of y-axes are labeled. Each data point is colored based on the total number of hits of this TF's motifs in the open chromatin regions. (C-D) DNA sequence motif enrichment in the open chromatin regions, ranked by relative motif enrichment scores (x-axes, see Methods for details) in between E13.5 stomach and E16.5 forestomach (C) or hindstomach (D). *N* = 3. Y-axes indicates the expression log fold change of these TFs. Top 20 TFs on each side of x-axes and top 10 TFs on each side of y-axes are labeled. Each data point is colored based on the total number of hits of this TF's motifs in the open chromatin regions.

Supplementary Figure 9

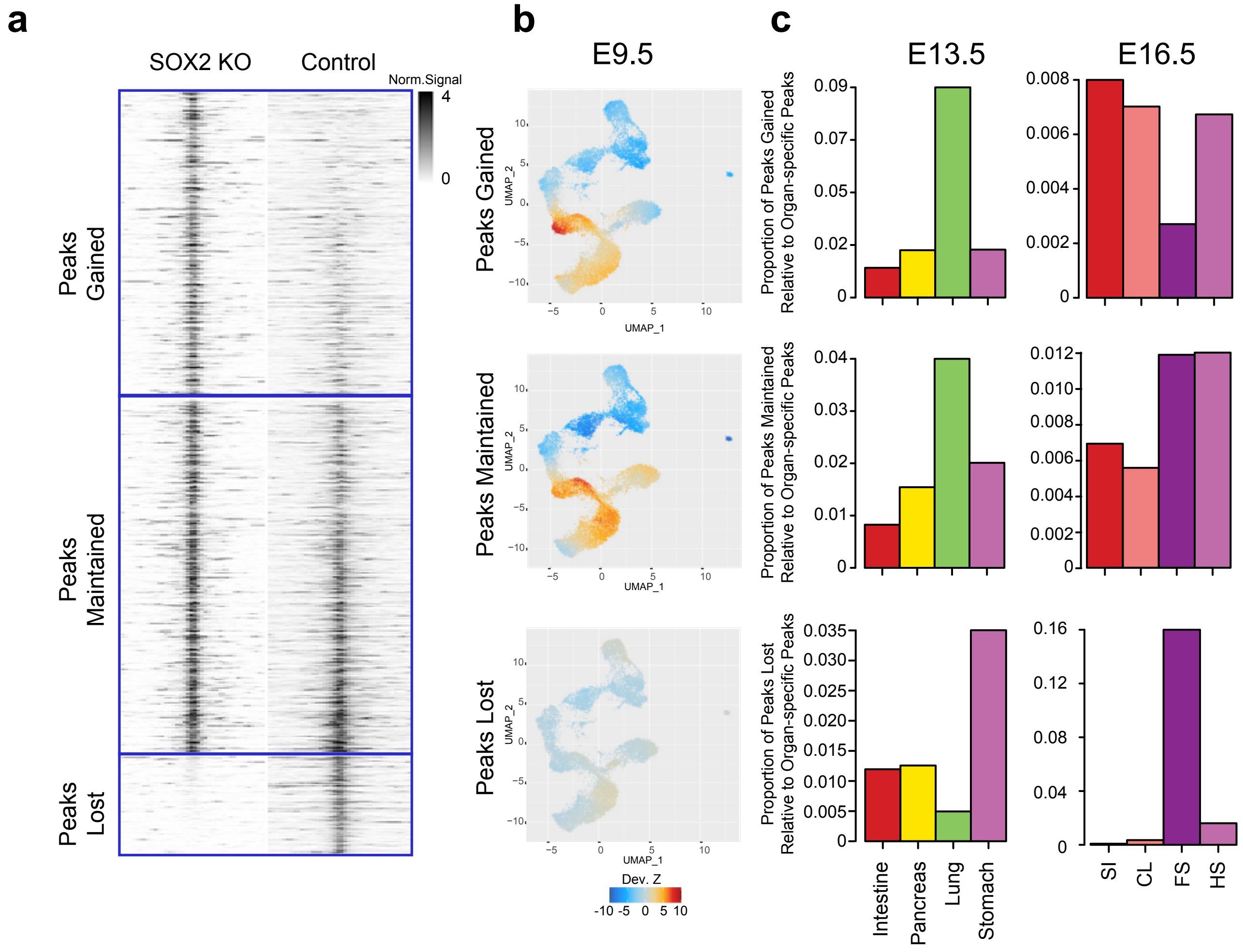

Figure S9. Sox2 loss is associated with changes in chromatin accessibility and gene expression. (A) ATAC-seq signal pattern around genome-wide chromatin accessibility peaks in E16.5 stomach from Sox2 KO (left) and control (right) samples. Peaks are grouped into three categories based on how chromatin accessibility changes upon Sox2 KO: Gained (top), Maintained (middle), and Lost (bottom). Grey scale represents normalized ATAC-seq signals. (B) Single cell scatter plots under the same UMAP representation of E9.5 scATAC-seq as Figure 1B, with each cell colored by its ChromVAR deviation Z-score for Sox2 KO Peaks Gained, Maintained, or Lost. (C) Proportion of chromatin accessibility peaks Gained, Maintained, or Lost upon Sox2 KO that are overlapped with E13.5 organ-specific chromatin accessibility peaks in the intestine, pancreas, lung, and stomach ($N = 7$, left) and with E16.5 organ-specific chromatin accessibility peaks in the small intestine (SI), colon (CL), forestomach (FS), and hindstomach (HS) ($N = 8$, right).

Supplementary Figure 10

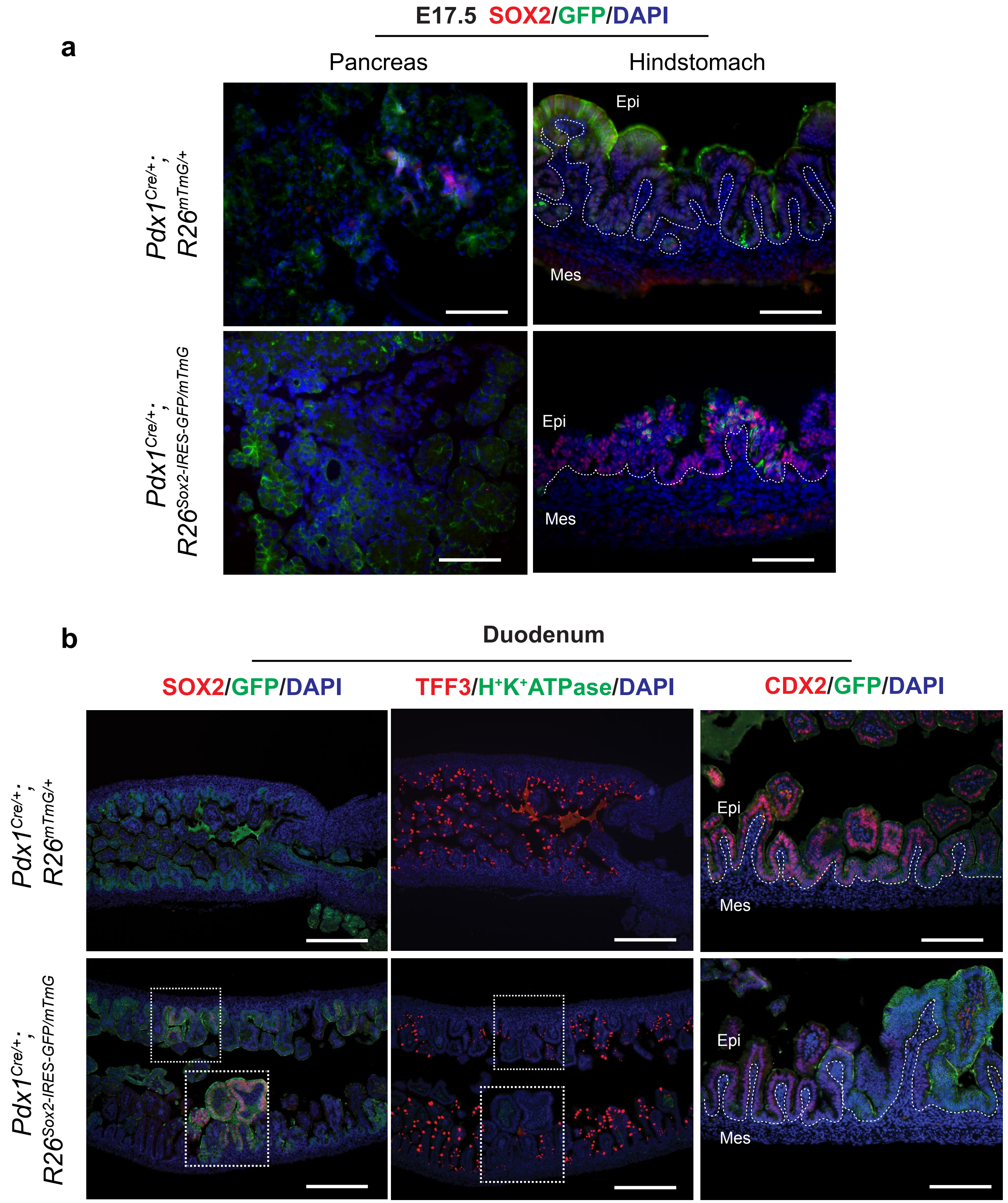

Figure S10. Sox2 overexpression has distinct effects on the pancreas and intestine. (A) Immunofluorescence analysis for SOX2 (red) and GFP (green) in E17.5 pancreas (left) and hindstomach tissues of Pdx1Cre/+;R26mTmG/+ (top) and Pdx1Cre/+;R26Sox2-IRES-GFP/mTmG (bottom) mice. Scale bars are 50μm. White dotted line separates the epithelial (Epi) and mesenchymal (mes) cells. (B) Immunofluorescence analysis for SOX2 (red) and GFP (green) (left), TFF3 (red) and H+K+ATPase (green) (middle), and CDX2 (red) and GFP (green) (right) in E17.5 Pdx1Cre/+;R26mTmG/+ top) and Pdx1Cre/+;R26Sox2-IRES-GFP/mTmG (bottom) duodenum samples. Scale bars for left and middle panels are 200μm, and 100μm for right panels. White dotted line separates the epithelial (Epi) and mesenchymal (Mes) cells.

Supplementary Figure 11

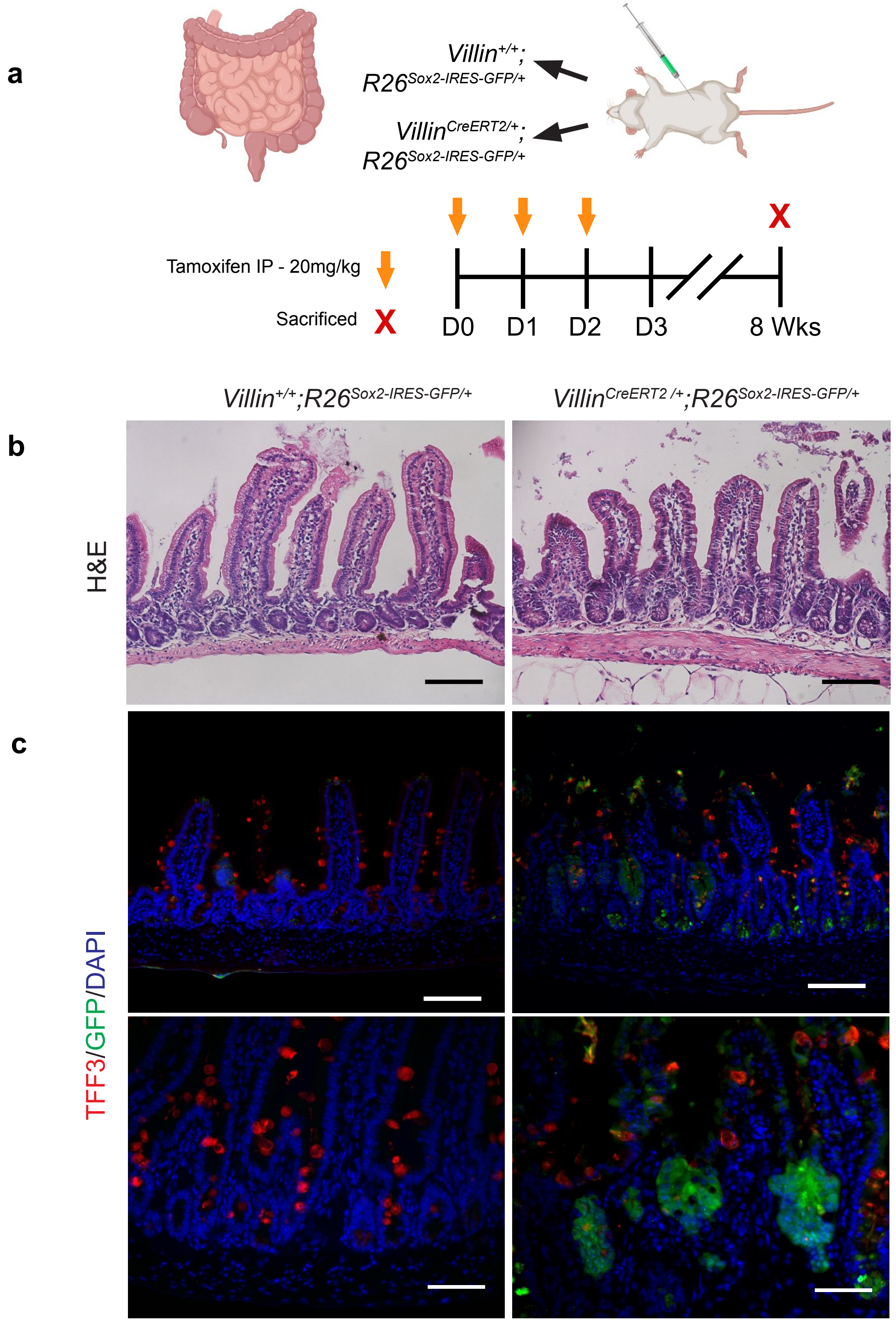

Figure S11. Sox2 overexpression alters cellular identity in adult intestinal epithelial cells. (A) Schematic overview of the tamoxifen treatment regime used to assess Sox2 overexpression in intestinal epithelial cells. (B) H&E stains of Villin+/+;R26Sox2-IRES-GFP/ + (left) VillinCreERT2/+;R26Sox2-IRES-GFP (right) intestinal samples. (C) Immunofluorescence analysis of TFF3 (red) and GFP (green) in Villin+/+;R26Sox2-IRES-GFP/ + (left) VillinCreERT2/+;R26Sox2-IRES-GFP (right) intestinal samples. Scale bars are 100μm in top panels and 50μm in bottom panels.

Supplementary Figure 12

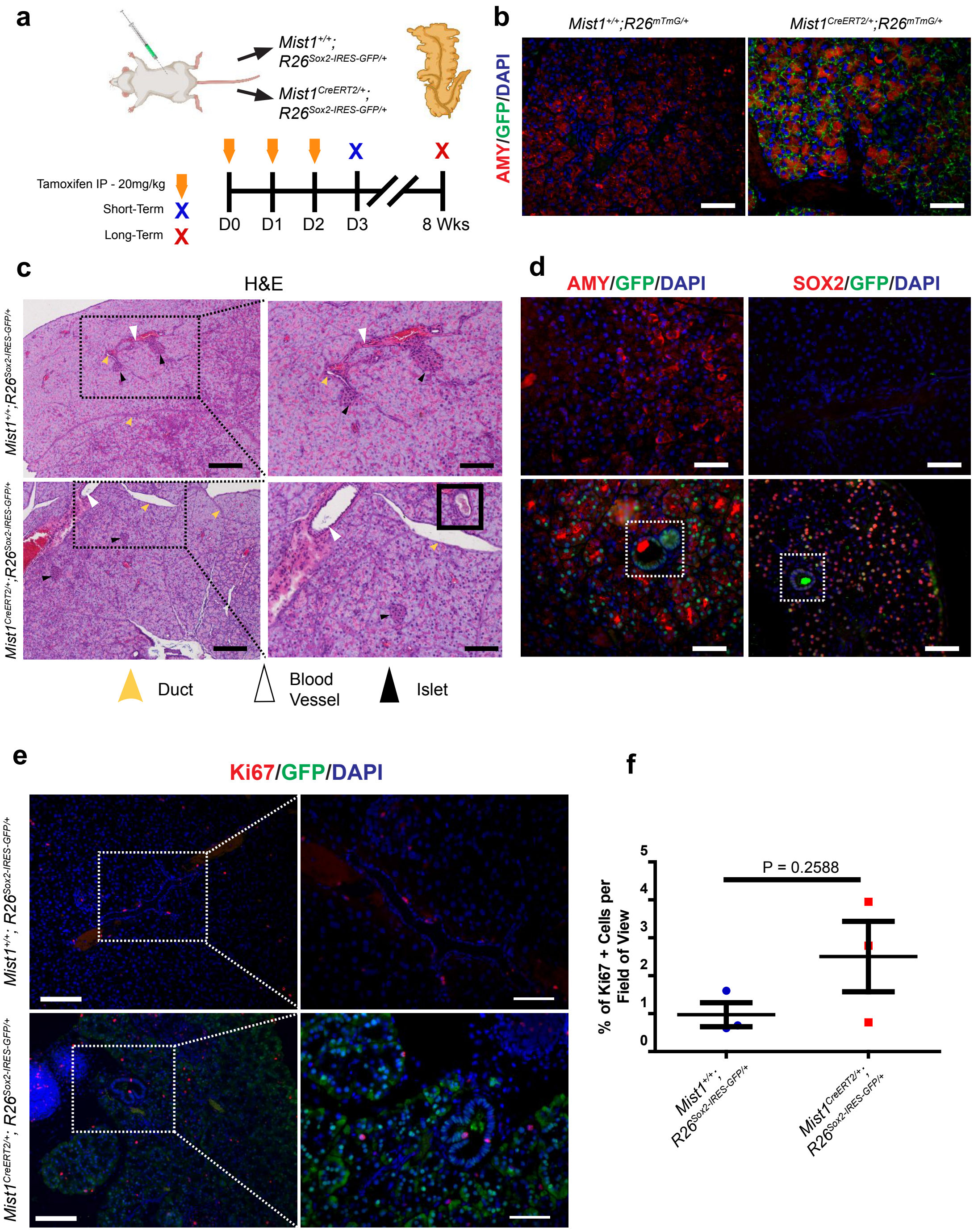

Figure S12. Short-term Sox2 overexpression in pancreatic acinar cells induces morphological changes in a small number of SOX2-expressing cells. (A) Diagram describing the tamoxifen treatment regime and sample harvesting schedule for SOX2 activation in acinar cells. (B) Short-term lineage-tracing analysis of Mist1 expression through immunofluorescence staining of Amylase (red) and GFP (green) in Mist1+/+;R26mTmG/+ (left) and Mist1CreERT2/+;R26mTmG/+ (right) pancreas tissues. Scale bars are 100µm. (C) H&E staining of Mist1+/+;R26Sox2-IRES-GFP/+ and Mist1CreERT2/+;R26Sox2-IRES-GFP/+ pancreas tissue. Black dotted boxes represent magnified area. Black arrowheads indicate pancreatic islets. White arrowheads point to blood vessels. Yellow arrowheads indicate pancreatic ducts. The black solid box outlines an abnormal ductal shape. Scale bars are 200µm (left panels) and 100µm (right panels). Immunofluorescence analysis of Mist1+/+;R26Sox2-IRES-GFP/+ (top) and Mist1CreERT2/+;R26Sox2-IRES-GFP/+ (bottom) pancreas tissue with (D) Amylase (red) and GFP (green) (left) and SOX2 (red) and GFP (green) (right). White boxes circular structures with GFP-expressing cells. Scale bars are 100µm. (F) Immunofluorescence analysis of Ki67 (red) and GFP (green) in Mist1+/+;R26Sox2-IRES-GFP/+ (top) and Mist1CreERT2/+;R26Sox2-IRES-GFP/+ (bottom) pancreas tissue. Scale bars are 100µm (left panels) and 50µm (right panels). White boxes indicate magnified area. (F) Mean (+ SEM) percent of proliferating cells marked by Ki67 in Mist1+/+;R26Sox2-IRES-GFP/+ and Mist1CreERT2/+;R26Sox2-IRES-GFP/+ samples (n=3 per genotype, NS = P>0.05, unpaired t-test with Welch's correction).

Supplementary Dataset 1. Quality control summary of biological replicates used in scATAC-seq datasets, related to Figure 1.

Supplementary Dataset 2. Enriched TF binding motifs identified in organ-specific clusters defined through scATAC-seq, related to Figure 1.

Supplementary Dataset 3. Number of genes which undergo changes in expression and chromatin accessibility from E9.5 to E13.5, related to Figure 2.

Supplementary Dataset 4. E18.5 *Cdx2* KO-specific and WT-specific gene lists, related to Figure 3.

Supplementary Dataset 5. Gene ontology analysis of E18.5 *Cdx2* KO-specific and WT-specific genes, related to Figure 3.

Supplementary Dataset 6. List of all genomics datasets used in the study with accession numbers.
