# Supplementary material for: Single-cell chromatin profiling of the primitive gut tube reveals regulatory dynamics underlying lineage fate decisions
Source: Nat Commun. 2022 May 26;13:2965. doi: 10.1038/s41467-022-30624-w (PMC9135761; doi:10.1038/s41467-022-30624-w)
Supplement: Supplementary file 2 — Description of additional Supplementary File [file 41467_2022_30624_MOESM2_ESM.pdf]

Descriptions of additional Supplementary data files

Supplementary Dataset 1. Quality control summary of biological replicates used in scATACseq datasets, related to Figure 1.

Supplementary Dataset 2. Enriched TF binding motifs identified in organ-specific clusters defined through scATAC-seq, related to Figure 1.

Supplementary Dataset 3. Number of genes which undergo changes in expression and chromatin accessibility from E9.5 to E13.5, related to Figure 2.

Supplementary Dataset 4. E18.5 Cdx2 KO-specific and WT-specific gene lists, related to Figure 3.

Supplementary Dataset 5. Gene ontology analysis of E18.5 Cdx2 KO-specific and WT-specific genes, related to Figure 3.

Supplementary Dataset 6. List of all genomics datasets used in the study with accession numbers.
