# Supplementary material for: Single-cell chromatin profiling of the primitive gut tube reveals regulatory dynamics underlying lineage fate decisions
Source: Nat Commun. 2022 May 26;13:2965. doi: 10.1038/s41467-022-30624-w (PMC9135761; doi:10.1038/s41467-022-30624-w)
Supplement: Supplementary file 9 — Reporting Summary [file 41467_2022_30624_MOESM9_ESM.pdf]

nature research

Corresponding author(s): Tae-Hee Kim and Chongzhi Zang

Last updated by author(s): Mar 8, 2022

Reporting Summary

Nature Research wishes to improve the reproducibility of the work that we publish. This form provides structure for consistency and transparency in reporting. For further information on Nature Research policies, see our Editorial Policies and the Editorial Policy Checklist.

Statistics

For all statistical analyses, confirm that the following items are present in the figure legend, table legend, main text, or Methods section.

n/a	Confirmed	
☐	☒	The exact sample size (*n*) for each experimental group/condition, given as a discrete number and unit of measurement
☐	☒	A statement on whether measurements were taken from distinct samples or whether the same sample was measured repeatedly
☐	☒	The statistical test(s) used AND whether they are one- or two-sided<br>*Only common tests should be described solely by name; describe more complex techniques in the Methods section.*
☐	☒	A description of all covariates tested
☐	☒	A description of any assumptions or corrections, such as tests of normality and adjustment for multiple comparisons
☐	☒	A full description of the statistical parameters including central tendency (e.g. means) or other basic estimates (e.g. regression coefficient) AND variation (e.g. standard deviation) or associated estimates of uncertainty (e.g. confidence intervals)
☐	☒	For null hypothesis testing, the test statistic (e.g. *F*, *t*, *r*) with confidence intervals, effect sizes, degrees of freedom and *P* value noted<br>*Give P values as exact values whenever suitable.*
☒	☐	For Bayesian analysis, information on the choice of priors and Markov chain Monte Carlo settings
☒	☐	For hierarchical and complex designs, identification of the appropriate level for tests and full reporting of outcomes
☒	☐	Estimates of effect sizes (e.g. Cohen's *d*, Pearson's *r*), indicating how they were calculated

Our web collection on statistics for biologists contains articles on many of the points above.

Software and code

Policy information about availability of computer code

Data collection	ImageJ (Version: Java 1.80_66) was used to process immunofluorescence image and to stitch whole-mount images of adult organs. FCS Express (version 7.04.00) was used to process flow cytometry data and generate flow cytometry plots.
Data analysis	A description of the software and code has been included in the Methods.<br>All computational code and scripts are available on GitHub at https://github.com/zang-lab/Single-cell-chromatin-profiling-of-the-primitive-gut-tube and Zenodo at https://doi.org/10.5281/zenodo.6394842.<br>HISAT2(2.1.0), HTSEQ(0.13.5), StringTie(2.1.4) and samtools(1.10) were used for bulk RNA-seq.<br>Bowtie2(2.4.0) and MACS2(2.2.7.1) were used for bulk ATAC-seq.<br>Bowtie2(2.4.0) and MACS1.4(1.4.3) were used for bulk ChIP-seq.<br>Seurat(3.2.2) was used for scRNA-seq.<br>Cellranger-ATAC(2.0.0) and ArchR(0.9.5) were used for scATAC-seq.<br>MEME suite(5.3.3) was used to identify TF motif sites.<br>limma(3.44.3) was used for microarray data analysis.<br>All the custom code is publicly available at https://github.com/zanglab/Single-cell-chromatin-profiling-of-the-primitive-gut-tube.

For manuscripts utilizing custom algorithms or software that are central to the research but not yet described in published literature, software must be made available to editors and reviewers. We strongly encourage code deposition in a community repository (e.g. GitHub). See the Nature Research guidelines for submitting code & software for further information.

Data

Policy information about availability of data

All manuscripts must include a data availability statement. This statement should provide the following information, where applicable:

- Accession codes, unique identifiers, or web links for publicly available datasets
- A list of figures that have associated raw data
- A description of any restrictions on data availability

Public data used in this study, including E9.5 single-cell RNA-seq data (GSE136689), ATAC-seq data of E13.5 intestine and stomach (GSE134275), E16.5 ATAC-seq data (GSE134275), E16.5 RNA-seq data (GSE134276), intestinal epithelium ATAC-seq data (GSE115314), CDX2 ChIP-seq data (GSE115314) and SOX2 ChIP-seq data (GSE134582) were downloaded from the Gene Expression Omnibus (GEO). Microarray data of E18.5 Cdx2-KO ileum and controls were downloaded from the European Bioinformatics Institute (EBI) ArrayExpress (accession numbers E-MTAB-92).
scATAC-seq, bulk ATAC-seq and RNA-seq from this study have been submitted to the NCBI Gene Expression Omnibus (GEO; http://www.ncbi.nlm.nih.gov/geo/) under accession numbers GSE168373.

scATAC-seq was used to generate figure 1B-E, 2B-E, 3E, 3G, S1B-D, S2A-C, S3A-D, S4A-D, S6A-D, S7A-D, and S9B.
E13.5 bulk ATAC-seq was used to generate figure 2A-F, 3A, 3F, 3G, S4A-D, S6A-D, S7A-C, S8A-D, and S9C.
E16.5 bulk ATAC-seq was used to generate figure 2F, 3D-I, S8A-D, and S9A-C.
scRNA-seq was used to generate figure 1C, 1E, 2C, 2D, S2B, S2C, S4A-D, and S7A-D
E13.5 bulk RNA-seq was used to generate figure 2C, 2D, S2B, S2C, S4A-D, S5, S7A-D, and S8A-D.
E16.5 bulk RNA-seq was used to generate figure S8A-D.
E18.5 bulk transcriptomic microarray was used to generate figure 3H and 3I.
ChIP-seq data was used to generate figure 3B, 3C, 3G, and 6B.

Field-specific reporting

Please select the one below that is the best fit for your research. If you are not sure, read the appropriate sections before making your selection.

☒ Life sciences ☐ Behavioural & social sciences ☐ Ecological, evolutionary & environmental sciences

For a reference copy of the document with all sections, see nature.com/documents/nr-reporting-summary-flat.pdf

Life sciences study design

All studies must disclose on these points even when the disclosure is negative.

Sample size	All sequencing was performed using two biological replicates. For image analysis and quantification, minimum three biological replicates were included. For mutant mouse strains, difficulties in acquiring mice of specific genotypes precluded calculations of sample size.
Data exclusions	No data were excluded from our analyses.
Replication	In vivo phenotypes were observed in all mutant mice (n=3 for each genotype), indicating reproducibility in our findings.
Randomization	Mice were allocated into experimental groups based on their genotype. Whenever possible, littermate controls were used to minimize variability due to differences in litters.
Blinding	Blinding was not relevant for sequencing analyses, as samples were all taken from untreated wildtype mice. E17.5 wholemount images and pancreatic mass measurements and data analysis were conducted prior to genotyping mice, so their identity was unknown at the the time of data collection and analysis. Similarly, quantification of proliferation was completed before mouse genotypes were known, so the researchers were blinded to their identity.

Reporting for specific materials, systems and methods

We require information from authors about some types of materials, experimental systems and methods used in many studies. Here, indicate whether each material, system or method listed is relevant to your study. If you are not sure if a list item applies to your research, read the appropriate section before selecting a response.

Materials & experimental systems

n/a	Involved in the study
	☒ Antibodies
☒	☐ Eukaryotic cell lines
☒	☐ Palaeontology and archaeology
	☒ Animals and other organisms
☒	☐ Human research participants
☒	☐ Clinical data
☒	☐ Dual use research of concern

Methods

n/a	Involved in the study
☒	☐ ChIP-seq
	☒ Flow cytometry
☒	☐ MRI-based neuroimaging

Antibodies

Antibodies used

For E9.5 endoderm isolation: 1:200 EPCAM (Abcam, ab95641)

Primary antibodies used for immunofluorescence:
SOX2 (Abcam, ab97959, 1:200),
GFP (Abcam, ab13970, 1:1000),
OLFM4 (Cell Signaling Technology, 39141S, 1:300),
CDX2 (Cell Signaling Technology, D11D10, 1:200),
Lysozyme C (DAKO, A0099, 1:1000),
PDX1 (DSHB F109-D12, 1:200),
Amylase (Abcam, ab21156, 1:500),
MUC1 (Invitrogen, MA5-11202, 1:300),
Ki67 (Abcam, ab15580, 1:500),
TFF3 (Gift from D. Podolsky, University of Texas Southwestern, Dallas, TX, 1:500) - Generated in the lab of D. Podolsky

Secondary antibodies used for immunofluorescence:
Alexa Fluor 568 (Invitrogen, A11011, 1:500),
Alexa Fluor 488 (Invitrogen, A11029, 1:500),
Alexa Fluor 488 (Invitrogen, A11039, 1:500),
Alexa Fluor 568 (Fisher Scientific, 127585160, 1:500)

Validation

Concentrations for immunofluorescence assays we optimized in our lab through serial dilution assays on control tissues.

Validation:

SOX2 (Abcam, ab97959, 1:200), Reactivty: mouse, Commercial test: ICC: NCCIT and NIH/3T3 cells. Dissociated induced pluripotent stem cells from mouse embryonic fibroblasts. Mouse embryonic stem cells.

GFP (Abcam, ab13970, 1:1000), reactivity: species independent, Commercial test: ICC: GFP-transfected NIH/3T3 (Mouse embryo fibroblast cell line)

OLFM4 (Cell Signaling Technology, 39141S (aka D6Y5A, 1:300), Reactivity: Mouse, Commercial Test: mouse small intestine using Olfm4 (D6Y5A) XP® Rabbit mAb (Mouse Specific).

CDX2 (Cell Signaling Technology, D11D10, 1:200), Reactivity: Human, published results in mouse: Kumar, N., Development (2019)

Lysozyme C (DAKO, A0099, 1:1000), Reactivity: Human, published results in mouse: Guiu, J., Nature (2019), Fenderico, Koren, E., Nat Commun. (2018), Bellono. N.W., Cell (2017), Yanai, H., Sci Rep (2017)

PDX1 (DSHB F109-D12, 1:200), Reactivity: Mouse, Rat, Commercial test: Starting concentration for immunohistochemistry (IHC), immunofluorescence (IF), and immunocytochemistry (ICC) when using mouse Ig is 2-5 ug/ml.

Amylase (Abcam, ab21156, 1:500), Reactivity: Mouse, human, Commercial verification statement: Our Abpromise guarantee covers the use of ab21156 in the following tested applications: Western Blot, immunohistochemistry – paraffin, immunohistochemistry - frozen.

MUC1 (Invitrogen, MA5-11202, 1:300), Reactivity: mouse, human, Commercial verification statement: This Antibody was verified by Relative expression to ensure that the antibody binds to the antigen stated

Ki67 (Abcam, ab15580, 1:500), Reactivity: Mouse, human, Commercial verification statement: Our Abpromise guarantee covers the use of ab15580 in the following tested applications: immunohistochemistry -paraffin, immunocytochemistry

Animals and other organisms

Policy information about studies involving animals; ARRIVE guidelines recommended for reporting animal research

Laboratory animals

Male and female mice (mus musculus) from mixed strain backgrounds (CD1, C57BL/6J mice) were harvested at E9.5, E13.5, E17.5,

Laboratory animals	and adult mice (either 6 weeks of age for short-term experiments or 14 weeks of age for long-term experiments). Mice were subject to a 12-hour day-night cycle and kept in ambient conditions of 21-23C with 40-60% humidity.
Wild animals	This study did not involve wild animals.
Field-collected samples	This study did not collect samples from the field.
Ethics oversight	All mice were handled in accordance with the rules and regulations of the Canadian Council on Animal Care Guidelines for Use of Animals in Research and Laboratory Animal Care under protocols approved by the Animal Care Committee at The Centre for Phenogenomics (protocol: 19-0276H).

Note that full information on the approval of the study protocol must also be provided in the manuscript.

Flow Cytometry

Plots

Confirm that:

☒ The axis labels state the marker and fluorochrome used (e.g. CD4-FITC).

☒ The axis scales are clearly visible. Include numbers along axes only for bottom left plot of group (a 'group' is an analysis of identical markers).

☒ All plots are contour plots with outliers or pseudocolor plots.

☒ A numerical value for number of cells or percentage (with statistics) is provided.

Methodology

Sample preparation	Single-cell ATAC-seq sample preparation:<br>For each biological replicate (n=2), E9.5 gut tubes were micro-dissected, washed in cold PBS and digested in 4ml of a 2:1 solution of Trypsin LE: 1x PBS at 37C. After 10 minutes, tissue was vigorously pipetted, until the organs broke down into a single-cell suspension, which was confirmed using brightfield microscopy. The enzymatic reaction was then inhibited with 10% FBS. After washing cells in cold PBS, the single cells were re-suspended in 400µl of 1:5000 Sytox Blue (ThermoFisher Scientific, S34857) and 1:200 EPCAM (Abcam, ab95641) in cold 1x PBS, filtered through a 30µm mesh and submitted for sorting.<br><br>Bulk RNA-seq and ATAC-seq preparation<br>Two biological replicates (n=2) at E13.5 were used experiments. ShhCre/+;R26mTmG/+ mice were used to isolate lung, stomach, and intestinal epithelial cells, while Pdx1Cre/+;R26mTmG/+ mice were used to isolate pancreatic epithelial cells. Tissues were prepared for sorting in the same manner as E9.5 samples but did not undergo EPCAM staining. Live, GFP-expressing cells were sorted.
Instrument	The Sony SH800 BRV instrument was used.
Software	FCS Express was used to analyze the data.
Cell population abundance	GFP and EPCAM are specifically expressed in endodermal cells in our population. All sorted cells were considered to be endodermal cells.
Gating strategy	Cells with low FSC and SSC were excluded, as these could be debris. The largest fraction of suitably sized cells was collected, excluding larger sizes which may represent doublets or clumps of undigested cells. Cells were considered viable by Sytox Blue when they were below a set threshold, as per the manufacturer's directions. Thresholds for GFP and EPCAM expression were determined through the use of GFP-negative and unstained EPCAM controls. Signal beyond this negative-control threshold was considered GFP-positive or EPCAM-positive.

☒ Tick this box to confirm that a figure exemplifying the gating strategy is provided in the Supplementary Information.
